# The Association between Malaria and Iron Status or Supplementation in Pregnancy: A Systematic Review and Meta-Analysis

**DOI:** 10.1371/journal.pone.0087743

**Published:** 2014-02-14

**Authors:** Laura Sangaré, Anna Maria van Eijk, Feiko O. ter Kuile, Judd Walson, Andy Stergachis

**Affiliations:** 1 Department of Global Health, University of Washington, Seattle, Washington, United States of America; 2 Department of Bioengineering, University of Washington, Seattle, Washington, United States of America; 3 Department of Epidemiology, University of Washington, Seattle, Washington, United States of America; 4 Department of Medicine and Department of Pediatrics, University of Washington, Seattle, Washington, United States of America; 5 Department of Clinical Sciences, Liverpool School of Tropical Medicine, Liverpool, United Kingdom; University of Copenhagen and Rigshospitalet, Copenhagen, Denmark

## Abstract

**Introduction:**

Malaria prevention and iron supplementation are associated with improved maternal and infant outcomes. However, evidence from studies in children suggests iron may adversely modify the risk of malaria. We reviewed the evidence in pregnancy of the association between malaria and markers of iron status, iron supplementation or parenteral treatment.

**Methods and Findings:**

We searched MEDLINE, EMBASE, the Cochrane Central Register of Controlled Trials, the Global Health Library, and the Malaria in Pregnancy library to identify studies that investigated the association between iron status, iron treatment or supplementation during pregnancy and malaria. Thirty one studies contributed to the analysis; 3 experimental and 28 observational studies. Iron supplementation was not associated with an increased risk of *P. falciparum* malaria during pregnancy or delivery in Africa (summary Relative Risk = 0.89, 95% Confidence Interval (CI) 0.66–1.20, I^2^ = 78.8%, 5 studies). One study in Asia reported an increased risk of *P. vivax* within 30 days of iron supplementation (e.g. adjusted Hazard Ratio = 1.75, 95% CI 1.14–2.70 for 1–15 days), but not after 60 days. Iron deficiency (based on ferritin and C-reactive protein) was associated with lower odds for malaria infection (summary Odds Ratio = 0.35, 0.24–0.51, I^2^ = 59.2%, 5 studies). With the exception of the acute phase protein ferritin, biomarkers of iron deficiency were generally not associated with malaria infection.

**Conclusions:**

Iron supplementation was associated with a temporal increase in *P vivax*, but not with an increased risk of *P. falciparum*; however, data are insufficient to rule out the potential for an increased risk of *P. falciparum*. Iron deficiency was associated with a decreased malaria risk in pregnancy only when measured with ferritin. Until there is more evidence, it is prudent to provide iron in combination with malaria prevention during pregnancy.

## Introduction

Anemia affects the lives of more than 500 million women in developing countries. The consequences of anemia during pregnancy include maternal mortality and stillbirth [1]–[5]. Iron deficiency is generally regarded as the most common cause of anemia accounting for an estimated 50% of all anemia worldwide. Iron deficiency anemia (IDA) is thought to cause an estimated 600,000 perinatal and 100,000 maternal deaths per year globally [6]. In areas where anemia is highly prevalent, international guidelines recommend universal iron and folic acid supplementation throughout pregnancy [7].

A recently updated Cochrane review showed evidence that pregnant women taking iron supplements reduced their risk on anemia by 70% (risk ratio [RR] 0.3, 95% confidence interval [CI] 0.19–0.46) and iron-deficiency at term by 57% (RR 0.43, 95% CI 0.27–0.66) [8]. In addition, they were less likely to have low birth weight newborns (<2500 grams) compared with controls (RR 0.81, 95% CI 0.68–0.97). The mean birth weight was 30.8 grams greater among infants whose mothers received iron during pregnancy (95% CI 5.9–55.7 grams), whereas the relationship between iron supplementation and premature birth (<37 weeks of gestation; RR 0.88, 95% 0.77–1.01) and neonatal death was not significant (RR 0.90, 95% CI 0.68–1.19) [8]. It is not clear yet if supplementation translates into clinical improvements such as reducing the incidence of puerperal infection or postpartum hemorrhage. Other benefits of maternal iron supplementation include improved newborn iron stores which are determined through in utero acquisition of iron [9], and these iron stores remain associated with the infants’ iron status at 9 and 24 months of age [10], [11]. This has important implications given that IDA has been associated with impaired cognitive and motor development in children [12], [13].

The benefits of maternal iron supplementation in malaria-endemic areas are less clear. An analysis of 101,636 singleton live-born infants conducted using data from Demographic and Health Surveys of 19 malaria-endemic countries in sub-Saharan Africa found that infants whose mothers received any iron/folic acid supplements and intermittent preventive treatment with sulfadoxine-pyrimethamine (IPTp-SP) for malaria during pregnancy were less likely to have a neonatal death compared to women who did not receive either iron/folic acid or malaria prophylaxis (hazard ratio (HR): 0.76; 95% CI 0.58–0.99) [14]. However, this effect was not seen among mothers who received only iron/folic acid supplements or only IPTp-SP. A recent meta-analysis of 32 nationally representative health surveys in sub-Saharan Africa, confirmed the protective association of malaria prevention (IPTp-SP and ITNs) with a protective efficacy of 18% on neonatal mortality (95% CI 4–30%) and of 21% for low birth weight (95% CI 14–27%) [15].

In summary, malaria prevention and iron supplementation are each associated with improved maternal and infant outcomes. However, the benefits of iron supplementation in pregnancy must be carefully weighed against the possibility of adverse consequences caused by this intervention in certain settings. Evidence from several studies among children suggests iron supplementation and iron status may adversely modify the risk of malaria, complicating a universal policy of routine iron supplementation in children in malaria endemic areas [16]–[19]. However, a Cochrane review of this topic concluded in the presence of regular malaria surveillance and appropriate treatment there is no increase in malaria risk among children [20]. A technical working group on iron and malaria established by the U.S. National Institute of Child Health and Human Development recently reviewed the evidence and concluded, “The balance of evidence indicates that the administration of iron supplements, usually in combination with folic acid, increases the risk of malarial morbidity when given without malarial prophylaxis, and in the absence of universal access to treatment” [21].

Despite the universal recommendation for iron supplementation in many malaria endemic countries, this question has yet to be examined in pregnancy, thereby creating uncertainty among public health programs regarding the use of iron supplementation during pregnancy in malaria endemic areas. To address this gap, we review the evidence in pregnancy of the association between the risk of malaria and iron status, iron treatment or iron supplementation.

## Methods

### Search Strategy

Studies investigating the association between iron status, iron treatment or iron supplementation during pregnancy and malaria risk were identified by searching MEDLINE, EMBASE, the Cochrane Central Register of Controlled Trials, the Global Health Library from the World Health Organization (WHO) and the Malaria in Pregnancy library from their inception to January 2013 inclusive without language restrictions [22]. The search was limited to human studies and used the following search terms: (pregnant OR pregnancy OR placental OR placenta OR fetus OR fetal OR foetus OR foetal) AND (iron OR ferrous OR ferric) AND (malaria OR parasitemia OR parasitaemia OR paludism OR plasmodium OR falciparum or vivax). The references of all identified articles, as well as additional review articles, were examined to locate additional studies not identified during the computerized search. Authors of potentially relevant articles were contacted if their methodology suggested relevant data were available but not presented in the published manuscript. The study adhered to the PRISMA statement [23].

### Selection

Using the search criteria defined above, 299 publications were identified. Each manuscript was reviewed by two authors (LS and AVE) and included if it met the following criteria: (1) study population included pregnant women; (2) data were available to assess the association between iron status, iron treatment, or iron supplementation and malaria; (3) for longitudinal studies and trials: an appropriate control group was included that did not receive iron; and (4) iron biomarkers were limited to serum ferritin, serum iron, serum transferrin, transferrin saturation (TS%), soluble transferrin receptor (sTfR), total iron binding capacity (TIBC), and erythrocyte protoporphyrin (EP). Discrepancies between reviewers were resolved through discussions until consensus was reached. For articles where it could be assumed that information was available but not presented in the format needed (e.g. the study reported on malaria and iron deficiency, but no data on iron deficiency by malaria status was presented), the authors were contacted for additional information.

### Validity Assessment

Details of the methods used to assess validity are included in the supplemental appendix. In summary, the Cochrane Collaboration’s tool for assessing the risk of bias among randomized trials was used to determine the quality of included trials as ‘low’ (high risk of bias), ‘high’ (low risk of bias), or ‘unclear’ [24]. The assessment of non-randomized study designs were based on source population, participant selection, completeness of exposure and outcome data, appropriate tests, sample size, and measures to control confounding [25], [26]. Quality was classified as low-to-moderate or good (Supplement 1, Figure S1.1, Figure S1.2 in File S1).

### Data Abstraction

A standardized data abstraction form was used to collect the following data elements: year of publication, geographic location, population, design, number of subjects enrolled, malaria endemicity, duration, frequency and dose of iron, concurrent malaria treatment or prevention, incidence or prevalence of malaria parasitemia or placental malaria infection and biomarkers for iron status.

### Classification of Studies

Studies focusing on iron status, iron treatment, and iron supplementation were considered separately. Iron status studies were further stratified into iron deficiency and iron biomarkers studies. Study design was classified as randomized-controlled trial (RCT), prospective cohort study, retrospective cohort study, case-control study, cross-sectional study, or before-after study.

### Outcomes

The primary outcomes of interest were peripheral malaria parasitemia during pregnancy or peripheral or placental malaria infection at the time of delivery by blood smear. Information from additional malaria tests were included where available (e.g. placental histology, polymerase chain reaction).

### Meta-analysis

Data from studies of iron deficiency status and individual iron biomarkers were summarized using forest plots and meta-analysis was conducted where possible. If malaria was assessed by more than one method, the most common test was utilized for the pooled analysis to decrease heterogeneity. Iron supplementation data were transformed into summary risk ratios (RR) and 95% confidence intervals (CI) estimated as the ratio of the proportion of women with malaria infection among those receiving and not-receiving iron supplementation during pregnancy. Available information in included studies allowed three subgroup analyses: by HIV status, by duration of iron supplementation, and by sickle cell genotype. Causes of anemia among HIV-infected pregnant women, iron status and effect of iron supplementation differ from HIV-negative pregnant women [27], [28].

Iron deficiency data was described as ratio of the odds of malaria among iron deficient vs. non-deficient pregnant women in cross-sectional studies and case-control studies. Pooled analyses of iron deficiency and malaria were stratified by timing of malaria test (during pregnancy or at delivery) and the definition of iron deficiency. We used three groups; 1) an iron-deficiency definition based on ferritin and C-reactive protein (CRP, e.g. ferritin <30 ng/mL with CRP< = 8·2 ng/mL or ferritin<70 ng/mL with CRP>8·2 ng/mL, the most common definition), 2) a definition based on ferritin alone or in another combination (e.g. sTfR/log ferritin ratio), and 3) all other definitions that did not use ferritin (e.g. erythrocyte protoporphyrin). The summary odds ratio (OR) and 95% CI were computed as the pooled ratio of the odds of malaria among iron deficient and iron replete women; where available, adjusted odds ratios were used.

Mean differences (MD) were computed to compare the overall effect for all individual biomarkers by transforming biomarkers to the same unit (serum iron, TIBC, sTfR, and TS). Because of skewed distributions for most of the ferritin data, we used geometric means for ferritin; studies which did not present geometric means were transformed and geometric mean difference and 95% confidence interval calculated using the Taylor series approximation [29], [30]. In addition, the ratios of geometric means were calculated. Individual biomarkers were not stratified by type of malaria test due to the similarity in results between peripheral and placental malaria. All meta-analyses were done using a DerSimonian and Laird random effects model [31]. The Cochrane’s chi-squared test for heterogeneity set at a significance of *p*<0.10 was evaluated. The extent of heterogeneity was measured using I^2^, a measure of the proportion of total variability explained by heterogeneity; this is expressed as a percentage, with 0–25% indicating no or little heterogeneity [32]. Data were analyzed using Stata 12.1 (Stata Corporation, College Station, TX), and Comprehensive Meta analysis (version 2.2.055) was used for subgroup analysis.

## Results

**Figure 1:**
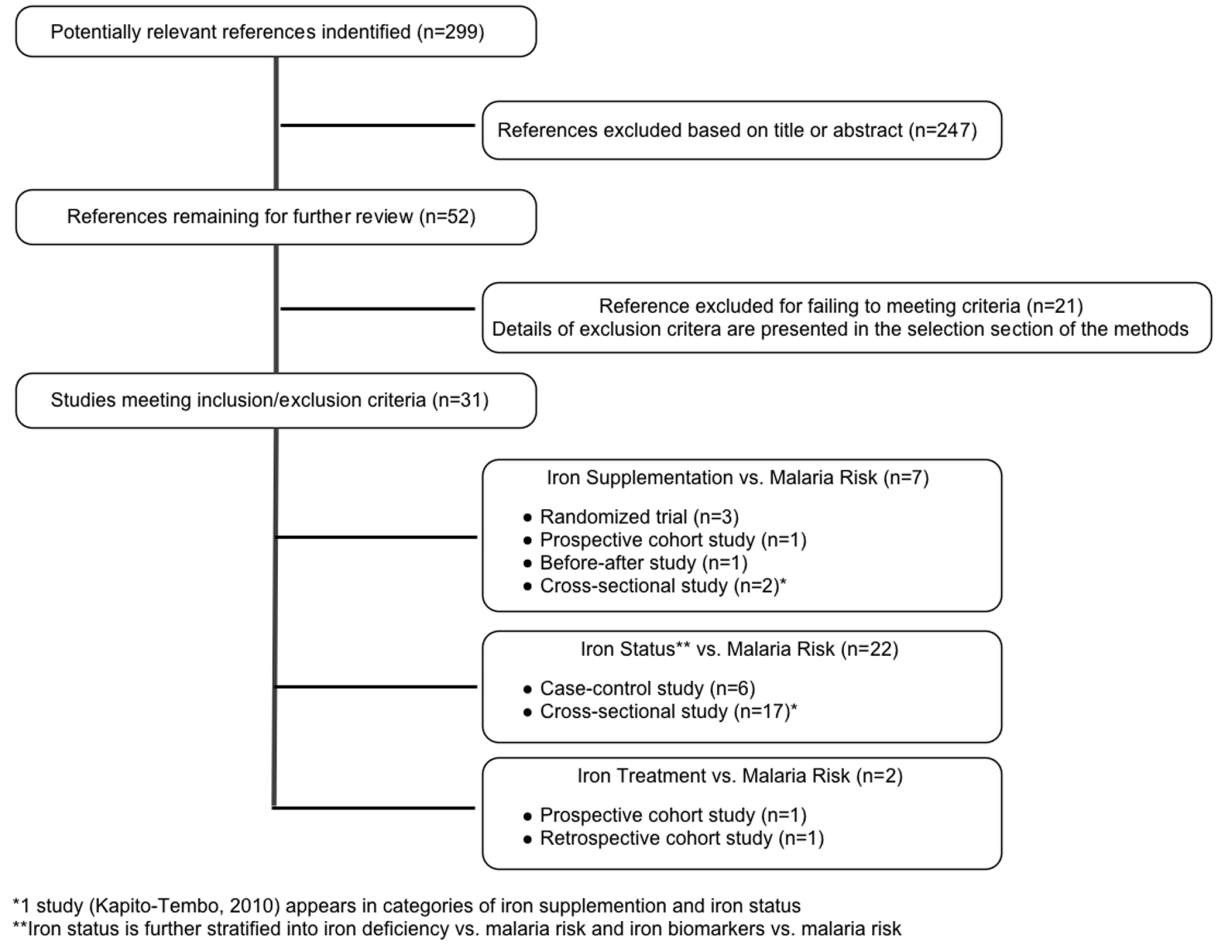
Flow diagram of study selection. Footnote: *1 study (Kapito-Tembo 2010) appears in categories of iron supplementation and iron status. **Iron status is further stratified into iron deficiency vs. malaria risk and iron biomarkers vs. malaria risk.

Of 299 published studies identified, 31 met the inclusion criteria (figure 1). Included studies were from a broad range of geographic locations and published between 1970 and 2012. Studies were categorized as those assessing the association between iron supplementation and malaria risk in pregnancy [33]–[39], iron deficiency and malaria risk in pregnancy [34], [40]–[50], iron biomarkers and malaria risk in pregnancy [40]–[43], [46], [47], [50]–[61], or iron treatment and malaria risk in pregnancy [62], [63]. These included two randomized controlled trials [36], [37] and one sub-group analysis [33] from the main trial [37], two prospective cohort studies [38], [62], one before-after study [39], one retrospective cohort study [63], six case-control studies [40], [47], [49], [52]–[54], and 18 cross-sectional studies (table 1–4) [34], [35], [41]–[46], [48], [50], [51], [55]–[61]. One cross-sectional study was described in two articles, the original analyzed serum ferritin relative to malaria infection [57] and the supplemental article considered iron deficiency status controlling for inflammation relative to malaria infection [45].

**Table 1:** Summary of included studies for iron supplementation and malaria risk in pregnancy.

Author, Year	Country (Time period)	Study Design	Population	Malaria endemicity (Study prevalence)	Iron Dose (and folic acid if available)	Concurrent malaria control or treatment	Iron supplementation	Comparison group (no iron supplementation)	Outcome
Kapito-Tembo 2010 [34]	Malawi (Dec 2005–July 2009)	Cross-sectional	HIV (+) pregnant women with ≥34 gestational wks attending routine ANC. Women <15 yrs and with immediate life-threatening medical and obstetric conditions were excluded	Endemicity not stated (10%: PCR; 5·5%: Microscopy)	Formulation not stated	49.7% IPTp-SP; 29.8% CTX; 15.4% IPTp+CTX; 59.6% bed net	1) Any iron use 2) duration iron use >30 days	1) No iron use 2) duration iron use ≤30 days	Peripheral parasitemia at enrollment
Menendez 1994 [37]	The Gambia (1980s)	RCT	Multigravida pregnant women excluding those with a packed cell volume <25% at either of the first 2 visits	Seasonal with high transmission (36 wks = 27%; Placental malaria = 57%)	200 mg ferrous sulphate daily ( = 60 mg elemental iron) daily distributed by TBAs to study participants on a weekly basis and 5 mg Folic acid weekly	Women with parasitemia were treated with CQ 25 mg/base/kg for 3 days	Iron	Placebo	1) Peripheral parasitemia;2) Placental malaria
Menendez 1995 [33]	The Gambia (1980s)	Subgroup analysis from RCT (Menendez, 1994)	Multigravida pregnant women excluding those with a packed cell volume <25% at either of the first 2 visits	Seasonal with high transmission (36 wks = 27%; Placental malaria = 57%)	200 mg ferrous sulphate daily ( = 60 mg elemental iron) daily distributed by TBAs to study participants on a weekly basis and 5 mg Folic acid weekly	Women with parasitemia were treated with CQ 25 mg/base/kg for 3 days	1) AA+Iron; 2) AS+Iron	1) AA+Placebo; 2) AS+Placebo	1) Peripheral parasitemia at 36 wks; 2) Postnatal parasitemia; 3) Placental malaria
Mwapasa 2004 [35]	Malawi, Dec 2000, June 2002)	Cross-sectional	Pregnant women attending the labor ward and were excluded if they were less than 15 years of age or had hypertension, multiple gestations or altered consciousness.	Perennial with peaks December to April (10·6%)	400mg iron +5 mg Folic acid daily	95% of women received 1+ dose of IPTp-SP; 23.2% used bed net	1) Iron+FA use 2) duration of Iron >30 days	1) No Iron+FA use 2) duration of iron use ≤30 days	Peripheral parasitemia at delivery
Nacher 2003 [38]	Thailand (1993–1997)	Prospective Cohort	Pregnant women in camps for displaced persons on the Thai-Burmese border excluding those with malaria during the index pregnancy before enrollment and women with malaria at enrollment or within 8 weeks of follow-up	Seasonal with EIR<1 (PV = 15%; PF = 5.7%)	If HCT<30%: 5 mg FA+600 mg Ferrous Sulfate daily until delivery	PV-CQ; PF-Quinine, MQ or Artesunate	Duration of FA+Iron: 1–15d 16–30d 31–60d >60d	No FA+ Iron use	First malaria episode during follow-up
Ndyomugyeny 2000 [36]	Uganda (Feb 1996–Feb 1998)	RCT	Primigravida in their first or second trimester attending ANC for the first time without severe anemia	Hyper-endemic(38.2% at enrollment; 39% at delivery)	120 mg elemental iron daily +5 mg folic acid weekly	Active case management (group A also received 300 mg CQ weekly)	Active case management+Iron/FA	Active case management+Placebo	1) Peripheral parasitemia; 2) Placental malaria
van Eijk 2007 [39]	Kenya (1996–2000)	Before-After study	Pregnant women with uncomplicated singleton pregnancies >32 weeks gestation excluding those with underlying chronic illness	(3^rd^ trimester malaria = 16.7%; Placental malaria = 16.7%)	200 mg ferrous sulphate 3 times per day +5 mg folic acid		Period 2: Hematinics Sept 97– Mar 99	Period 1 - no intervention before Sept 1997	1) Peripheral parasitemia in 3^rd^ trimester; 2) Placental malaria

AA: Hemoglobin genotype AA. AS: Hemoglobin genotype AS. ANC: antenatal clinic. CQ: Chloroquine. CTX: Cotrimoxazole. D: days. EIR: Entomologic inoculation rate (# infectious bites/person/year). FA: Folic Acid. Hct: hematocrit. IPTp-SP: Intermittent presumptive treatment in pregnancy with sulfadoxine-pyrimethmine. ITN: Insecticide treated bed net. MQ: Mefloquine. PCR: polymerase chain reaction used to detect malaria. PF: *Plasmodium falciparum*. PV: *Plasmodium vivax.* RCT: randomized controlled trial.

**Table 2:** Summary of included studies for iron deficiency and malaria risk in pregnancy.

Author, Year	Country (Time period)	Study Design	Population	Malaria endemicity (Study prevalence)	Iron Dose (and folic acid if available)	Concurrent malaria control or treatment	Exposure: Malaria	Comparison: no malaria	Outcome
Abrams 2005 [40] a	Malawi (Feb-Oct 2002)	Case-control	Pregnant women attending the labor ward who did not have HIV, preeclampsia or multiple gestations	Perennial with peaks December to April (12.7%)	Not stated	91% took antimalarial tablets; 26% slept under mosquito nets	Peripheral parasitemia at delivery	No peripheral parasitemia at delivery	Iron deficiency^b^ at delivery
Dreyfuss 2000 [46]	Nepal (Aug 1994–Mar 1997)	Cross-sectional	Pregnant women 15–40 y from the placebo arm of an RCT	Hyper-endemic (*P. vivax*: 19.8%)	Not stated	Not stated	Peripheral parasitemia (*P. vivax*)	No peripheral parasitemia	Iron deficiency^c^
Engmann 2008 [41] d	Ghana (May-Aug, 2003)	Cross-sectional	Pregnant women 18–40 yrs with singleton pregnancies receiving ANC. Women with sickle cell, major congenital or current illnesses were excluded.	Endemicity not stated (7%)	All patients attending ANC receive free iron supplementation (formulation not stated)	All patients attending ANC receive IPTp	Peripheral parasitemia at enrollment	No peripheral parasitemia at enrollment	Iron deficiency^e^ at enrollment
Hinderaker 2002 [47] a	Tanzania (Feb 1995–Mar 1996)	Case-Control^f^	Pregnant women at their first ANC visit	Endemicity not stated (18.1%)	NA	NA	Peripheral parasitemia at enrolment	No peripheral parasitemia at enrollment	Iron deficiency^b^ at enrollment
Matteelli 1994 [42]	Zanzibar (Dec 1989–Apr 1990)	Cross-sectional	Pregnant women admitted for uncomplicated delivery	Highly endemic (21.5%)	Not stated	Not stated	Peripheral parasitemia at delivery	No peripheral parasitemia at delivery	Iron deficiency^g^ at enrollment
Ouédraogo 2012 [43] a	Benin (Jan 2010–May 2011)	Cross-sectional	HIV (-) pregnant women with <28 gestational wks attending routine ANC who had not yet taken IPTp, iron, folic acid, vitamin B12 or anti-helminthics	Perennial with two high seasonal peaks (15.1%)	NA	NA	Peripheral parasitemia at enrolment	No peripheral parasitemia at enrollment	Iron deficiency^b^ at enrollment
Senga 2011 [49] a	Malawi (2004–2005)	Case-Control	All pregnant women who attended the hospital for delivery excluding those with emergency obstetric conditions	Highly endemic with year-round transmission and seasonal peaks	60 mg Iron +5 mg Folic acid daily mostly in the 2^nd^ half of pregnancy	95% of women received 1+ doses of IPTp-SP	Placental malaria	No placental malaria	Iron deficiency^h^ at delivery
Van Santen 2011 [50] a	Gabon (2000–2004)	Cross-sectional	Primigravida without peripheral parasitemia at enrollment with singleton pregnancy with no indication of systemic infection	Stable meso - hyperendemic	60 mg iron daily provided through ANC	Not stated	Placental Malaria	No Placental Malaria	Iron deficiency^b^ at delivery
Danquah 2008 [45]	Ghana (1998)	Cross-sectional	Pregnant women presenting for routine ANC	Holoendemic (63%)	NA	NA	Iron deficiency at enrollment^c^	Iron replete at enrollment	Peripheral PF parasitemia at enrollment
Kabyemela 2008 [48]	Tanzania (2002–2005)	Cross-sectional	Participants of the Mother-Offspring Malaria Studies Project in Tanzania excluding those with evidence of chronic or debilitating illnesses	Intense malaria transmission EIR ?400 (12.4%)	Not stated	56·8% of women used IPT; ITNs were used by 14·7% of women	Iron deficient at delivery^c^	Iron replete at delivery	Placental malaria
Kapito-Tembo 2010 [34]	Malawi (Dec 2005–July 2009)	Cross-sectional	HIV (+) pregnant women with ≥34 gestational wks attending routine ANC. Women <15 yrs and with immediate life-threatening medical and obstetric conditions were excluded	Endemicity not stated (10%: PCR; 5.5%: Microscopy)	Formulation not stated	49.7% IPTp-SP; 29.8% CTX; 15.4% IPTp+CTX; 59.6% bed net	Iron deficiency^c^	No iron deficiency	Peripheral parasitemia at enrollment
Senga 2012 [44]	Malawi (1992–1995)	Cross-sectional	Pregnant women at first ANC visit and at delivery participating in a cohort study to assess effect MiP on fetal hemoglobin	Highly endemic with year-round transmission and seasonal peaks	60 mg iron and 250 μg folic acid daily through ANC	69.1% at delivery at least one dose of SP	Iron deficiency^i^	No iron deficiency	Peripheral parasitemia at enrollment Peripheral and placental malaria at delivery

ANC: antenatal clinic. CQ: Chloroquine. CTX: Cotrimoxazole. EIR: Entomologic inoculation rate (# infectious bites/person/year). IPTp-SP: Intermittent presumptive treatment in pregnancy with sulfadoxine-pyrimethmine. ITN: Insecticide treated bed net. MiP: malaria in pregnancy. MQ: Mefloquine. PF: *Plasmodium falciparum*. PV: *Plasmodium vivax.*

a Author contacted and additional information was obtained.

b Ferritin <30 ng/mL with CRP< = 8.2 ng/mL or ferritin<70 ng/mL with CRP>8.2 ng/mL.

c Serum ferritin <10 (μg/L) or Erthyrocyte protoporphyrin >70 (μmol/mol heme).

d Author contacted and author responded, but no additional information was available.

e Serum ferritin ≤16 ng/mL.

f Selected based on hemoglobin status (<70 g/L, 70–90 g/L, 90–110 g/L, 110–150, and >150).

g Serum ferritin ≤15 ng/mL.

h sTfR:log ferritin ratio >1.6.

i red cell zinc protoporhyrin/heme >2.7 μg/g hemoglobin.

**Table 3:** Summary of included studies for iron biomarkers and malaria risk in pregnancy.

Author, Year	Country (Time period)	Study Design	Population	Malaria endemicity (Study prevalence)	Iron Dose (and folic acid if available)	Concurrent malaria control or treatment	Malaria Infection	Comparisons group: No malaria infection	Outcome	
Abrams 2005 [40] a	Malawi (Feb-Oct 2002)	Case-control	Pregnant women attending the labor ward who did not have HIV, preeclampsia or multiple gestations	Perennial with peaks December to April (12.7%)	Not stated	91% took antimalarial tablets; 26% slept under mosquito nets	Peripheral parasitemia at delivery	No peripheral parasitemia	Iron biomarkers^b^ at delivery	
Asaolu 2009 [53]	Nigeria (Not stated)	Case-control	Pregnant women attending ANC	Endemicity not stated (Unclear if selected on malaria status)	Not stated	Not stated	Peripheral parasitemia at enrolment	No peripheral parasitemia at enrollment	Serum iron (μmol/L)	
Ayoya 2006 [51]	Mali (June-Aug 2002)	Cross-sectional	Pregnant women (18–45 yrs) attending community health clinic during study period excluding those who used oral iron or antihelminthics since the start of pregnancy or those with a blood transfusion in the 3 months before study entry	Highly endemic (11%)	NA	NA	Peripheral PF parasitemia at enrollment	No peripheral PF parasitemia at enrollment	Iron biomarkers^c^ at enrollment	
Dreyfuss 2000 [46]	Nepal (Aug 1994–Mar 1997)	Cross-sectional	Pregnant women 15–40 y from the placebo arm of an RCT	Hyper-endemic (*P. vivax*: 19.8%)	Not stated	Not stated	Peripheral parasitemia (*P. vivax*)	No peripheral parasitemia	Serum ferritin (μg/L)	
Engmann 2008 [41] d	Ghana (May-Aug, 2003)	Cross-sectional	Pregnant women 18–40 yrs with singleton pregnancies receiving ANC. Women with sickle cell, major congenital or current illnesses were excluded.	Endemicity not stated (7%)	All patients attending ANC receive free iron supplementation (formulation not stated)	All patients attending ANC receive IPTp	Peripheral parasitemia at enrollment	No peripheral parasitemia at enrollment	Serum ferritin (μg/L) at enrollment	
Eteng 2010 [54]	Nigeria (before 2010)	Case-control	Pregnant women attending ANC with symptomatic malaria and healthy controls. Women were included if they were not on iron therapy or hematinic drugs	Malaria endemic (NA-selected on malaria status)	None used	Not stated	Peripheral parasitemia at enrollment	No peripheral parasitemia at enrollment	Iron biomarkers^e^ at enrollment	
Hinderaker 2002 [47] a	Tanzania (Feb 1995–Mar 1996)	Case-Control^f^	Pregnant women at their first ANC visit	Endemicity not stated (22.8%)	NA	NA	Peripheral parasitemia at enrolment	No peripheral parasitemia at enrollment	Iron biomarkers^g^ at enrollment	
Huddle 1999 [55]	Malawi (Nov 1993–Feb 1994)	Cross-sectional	Pregnant women attending ANC aged 14–45 y with no history of c-section and hb>80 g/l	Endemicity not stated (31%)	Not stated	Not stated	Peripheral parasitemia	No peripheral parasitemia	Iron biomarkers^h^	
Massawe 2002 [56] d	Tanzania (Aug-Sept 1998)	Cross-sectional	Consecutive primigravida women ≤20 yrs attending their first ANC visit	Not stated (43.4%)	NA	NA	Peripheral parasitemia at enrollment	No peripheral parasitemia at enrollment	Iron biomarkers^i^ at enrollment	
Matteelli 1994 [42]	Zanzibar (Dec 1989–Apr 1990)	Cross-sectional	Pregnant women admitted for uncomplicated delivery	Highly endemic (21.5%)	Not stated	Not stated	Peripheral parasitemia at delivery	No peripheral parasitemia at delivery	Serum ferritin (ng/mL)	
Mockenhaupt 2000 [57]	Ghana (Nov-Dec 1998)	Cross-sectional	Pregnant women attend ANC	Endemicity not stated (32%)	Not stated	Not stated	Peripheral parasitemia at enrollment	No peripheral parasitemia at enrollment	Serum ferritin (ng/mL)	
Ndyomugyenyi 2008 [61] d	Uganda (2003–2004)	Cross-sectional	Pregnant women >16weeks at first ANC	Hyperendemic (35%)	NA	NA	Parasitemia at enrollment	No parasitemia at enrollment	Serum ferritin (μg/L) at enrollment	
Ouédraogo 2012 [43] a	Benin (Jan 2010–May 2011)	Cross-sectional	HIV (-) pregnant women with <28 gestational wks attending routine ANC who had not yet taken IPTp, iron, folic acid, vitamin B12 or anti-helminthics	Perennial with two high seasonal peaks (15.1%)	NA	NA	Peripheral parasitemia at enrolment	No peripheral parasitemia at enrollment		Serum ferritin (μg/L) at enrollment
Reinhardt 1978 [58]	Ivory Coast (Not stated)	Cross-sectional	Women with singleton deliveries	Endemicity not stated (39.4%; Peripheral parasitemia: 32.8%)	Not stated	Not stated	Peripheral parasitemia or placental malaria	Neither peripheral parasitemia or placental malaria	Iron biomarkers^j^ at delivery	
Saad 2012 [52]	Sudan (Aug-Dec 2010)	Case-control	Control group consisted of pregnant women with uncomplicated malaria and healthy pregnant women	Malaria endemic (NA-selected on severe malaria status)	NA	NA	Peripheral parasitemia at enrolment 1) Uncomplicated malaria; 2) Severe malaria	No peripheral parasitemia at enrollment	Serum ferritin(μg/L) at enrollment	
Shulman 1996 [59]	Kenya (Nov 1993)	Cross-sectional	Pregnant women attending ANC	Perennial transmission EIR 10 (23.6%)	Not stated	Not stated	Peripheral parasitemia at enrolment	No peripheral parasitemia at enrollment	Serum ferritin (ng/mL)	
VanderJagt 2007 [60] a	Nigeria (June-Aug 2003)	Cross-sectional	Healthy normotensive pregnant women with no history of hypertension, proteinuria or other complications of pregnancy	Endemicity not stated (9.4%)	Iron and folate supplements are provided to women at ANC whose hematocrit is indicative of anemia	25% of women took malaria prophylaxis	Peripheral parasitemia at enrolment	No peripheral parasitemia at enrollment	Iron biomarkers^k^	
Van Santen 2011 [50] a	Gabon (2000–2004)	Cross-sectional	Primigravida without peripheral parasitemia at enrollment with singleton pregnancy with no indication of systemic infection	Stable meso - hyperendemic	60 mg iron daily provided through ANC	Not stated	Placental Malaria	No Placental Malaria	Iron biomarkers^l^ at delivery	

ANC: antenatal clinic. CQ: Chloroquine. CTX: Cotrimoxazole. EIR: Entomologic inoculation rate (# infectious bites/person/year). IPTp-SP: Intermittent presumptive treatment in pregnancy with sulfadoxine-pyrimethmine. ITN: Insecticide treated bed net. MiP: malaria in pregnancy. RCT: Randomized Controlled Trial.

a Author contacted and additional information was obtained.

b sTfR (μg/mL) and ferritin (ng/mL).

c Serum iron <12 μmol/L; Mean Serum Iron; Mean TIBC.

d Author contacted and author responded, but no additional information was available.

e Transferrin (g/L); TIBC (μmol/L); Serum Iron (μmol/L); TS (%).

f Selected based on hemoglobin status (<70 g/L, 70–90 g/L, 90–110 g/L, 110–150, and >150).

g Serum iron (μmol/L); Serum ferritin (μg/L); TIBC (μmol/L); Transferrin saturation (%).

h Serum iron (μmol/L); TS (%); Serum ferritin (μg/L); sTfR (mg/L).

i Serum ferritin (μg/L) sTfR (mg/L).

j Serum iron (μg/100 mL); Transferrin (mg/100 mL).

k Serum iron (μg/dL); Serum ferritin (ng/mL); TIBC (pg/mL).

l Iron (μmol/L); TIBC (μmol/L); TS (%); Ferritin (μg/L); sTfR (mg/L).

**Table 4:** Summary of included studies for parenteral iron treatment and malaria risk in pregnancy.

Author, Year	Country (Time period)	Study Design	Population	Malaria endemicity (Study prevalence)	Iron Dose (and folic acid if available)	Concurrent malaria control or treatment	Intervention group A	Comparison group B	Outcome
Byles 1970 [62]	Tanzania	Prospective Cohort	Pregnancies <36 wks with Hb of <50% (<7.4 g/L) and pregnancies with >36 wks with Hb <60% were admitted to hospital for tx	Not stated	Single dose of iron dextran (mg of elemental iron = 0.66 (x) Weight (x) %Hb deficit)	300 mg CQ	Single dose iron dextran+Concurrent CQ	Single dose iron+No concurrent CQ	PF present only after infusion among patients with generalized reactions
Oppenheimer 1986 [63]	Papua New Guinea (1980–1981)	Retrospective Cohort	Pregnant women delivering in Madang provincial hospital	Intense malaria transmission with high incidence of CQ resistance	Single dose of 35 cc iron dextran = 1750 mg elemental iron provided at some time in the ANC period		Total Dose Iron Infusion	No Total Dose Iron Infusion	Post-natal malaria

ANC: Antenatal Clinic. CQ: Chloroquine. Hb: Hemoglobin. PF: *Plasmodium falciparum*. Tx: treatment.

### Quality of the Included Studies

The two trials, both published before the more widespread adoption of the CONSORT guidelines, were assessed as low quality, mainly because allocation concealment was not described. Overall, there were ten observational studies among the 25 evaluated which we marked as good quality. None of the observational studies reported a justification for the sample size of the study. For 16 studies, the association between iron and malaria was reported as one of the primary purposes of the analysis. The lack of a description of methods used to adjust for potential confounding was the most common deficit in the reporting of observational studies (15 times, eSupplement 1).

### Oral Iron Supplementation and Malaria Risk

#### P. falciparum malaria

The effect of iron supplementation on malaria risk in pregnancy was assessed in seven studies which included the two trials [36], [37], the one randomized trial sub-group analysis [33], one prospective cohort study [38], one before-after study [39], and two cross-sectional studies [34], [35]. The dose and duration of iron supplementation varied in the studies, as did the level of malaria endemicity and use of malaria prevention interventions (table 1).

**Figure 2:**
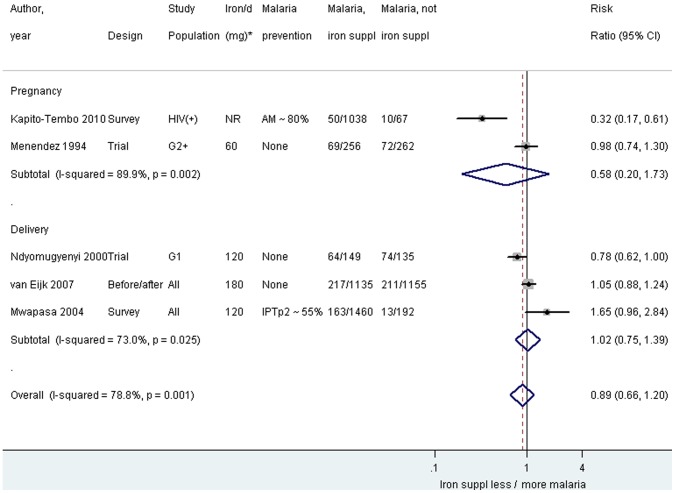
*Plasmodium falciparum* by blood smear among iron-supplemented and non-supplemented pregnant women by timing of malaria test. Footnote: AM: antimalarials used (either IPTp or cotrimoxazole). CI: confidence interval. G1: primigravidae. G2+: Multigravidae. HIV(+): HIV-positive. IPTp2: intermittent preventive treatment with 2 doses of sulfadoxine-pyrimethamine. *Daily dose of elementary iron. Notes: At delivery: placental blood smear for Ndyomugyenyi 2000 and van Eijk 2007, and peripheral blood smear for Mwapasa 2004. The weight for each study is indicated as a grey block around the risk estimate. Subgroup analyses and additional data are included in eSupplement 3.

A meta-analysis was performed among the iron supplementation studies stratified by timing of malaria test. This analysis excluded the sub-group analysis from the RCT because these women were represented in the parent study [33], and the study using hazard ratios because these data were only presented by duration of iron supplementation and therefore not comparable to the other studies [38]. The overall pooled result of iron supplementation during pregnancy or delivery was not associated with an increased risk of malaria (RR_pooled_ 0.89, 95% CI 0.66–1.20; I^2^ 78.8%, figure 2). Stratum specific results also showed no increase in risk of malaria associated with iron supplementation during pregnancy (RR_pooled_ 0.58, 95% CI 0.20–1.73; I^2^ 89.9%) or at the time of delivery (RR_pooled_ 1.02, 95% CI 0.75–1.39; I^2^ 73.0%, figure 2). Baseline iron status was not assessed in any of the studies, so a subgroup analysis among iron-replete and iron deficient women was not possible.

The results of further sub-group analysis can be seen in the supplement (File S1). No significant difference was seen by HIV status for the association between iron supplementation and the risk of malaria (p = 0.6 comparing groups by HIV-status, Figure S2.1 in File S1). Sub-group analyses from the randomized trial stratifying the data by hemoglobin genotype suggested a possible difference in placental malaria associated with iron supplementation by sickle cell genotype, however, this difference was not statistically significant (Figure S2.2 in File S1). Three studies (two surveys and a cohort) presented information by duration of iron supplementation. In a subgroup analysis for the two surveys in Africa, a duration of iron supplementation of >30 days was associated with a higher malaria risk (RR_pooled_ 1.42, 95% CI 1·09–1.84; I^2^ 0%, Figure S2.3 in File S1), despite high coverage of antimalarial prevention [34], [35]. In the survey among HIV-infected women, overall 65% used IPTp, 45% used daily cotrimoxazole prophylaxis (which has antimalarial activity), and 60% used nets; use of antimalarial prevention was less among women who did not use iron [34]. In the other survey, 94% of women used at least one dose of SP, and 23% used nets [35]. By contrast, the cohort study conducted on the Thai-Burmese border suggested no increase in risk of first episode of *P. falciparum* associated with iron supplementation up to 60 days (e.g. 1–15 days iron supplementation: Adjusted Hazard Ratio (aHR) 1.30, 95% CI 0.67–2.50, p = 0.4, table 5). Longer supplementation was even associated with a reduced risk (aHR iron supplementation >60 days: 0.37, 95% CI 0.17–0.77, p = 0.009, table 5). No antimalarial prophylaxis was used in this cohort and there was no information on net use but women were screened for malaria weekly and treated promptly [38].

**Table 5:** Summary of study outcomes – Iron Supplementation vs. Malaria Risk.

Author, Year	Study Design	HIV Status	Type & Timing of malaria test	Exposure	Comparisonto exposure	Risk estimate(95% CI)
**Outcome: First malaria episode during follow-up** – *Plasmodium vivax*						
				Iron+FA 1–15 d	No FA+ Iron use	aHR^1^ 1.75 (1.14, 2.70)
Nacher 2003 [38]	Prospective Cohort	All	Peripheral BS during pregnancy	Iron+FA 16–30d	No FA+ Iron use	aHR^1^ 1.70 (1.10, 2.60)
				Iron+FA 31–60d	No FA+ Iron use	aHR^1^ 1.30 (0.94, 1.80)
				Iron+FA >60d	No FA+ Iron use	aHR^1^ 0.67 (0.40, 1.02)
**Outcome: First malaria episode during follow-up** – *Plasmodium falciparum*						
				Iron+FA 1–15 d	No FA+ Iron use	aHR^1^ 1.30 (0.67, 2.50)
Nacher 2003 [38]	Prospective Cohort	All	Peripheral BS during pregnancy	Iron+FA 16–30d	No FA+ Iron use	aHR^1^ 0.90 (0.40, 2.10)
				Iron+FA 31–60d	No FA+ Iron use	aHR^1^ 1.40 (0.80, 2.40)
				Iron+FA >60d	No FA+ Iron use	aHR^1^ 0.37 (0.17, 0.77)

aHR: adjusted Hazard Ratio. BS: Blood slide. FA: Folic Acid.

1 Adjusted for *Plasmodium falciparum* or *Plasmodium vivax*, gravidity, age, estimated gestational age, past mean hematocrit.

#### *P. vivax* malaria

In the single study from Asia that assessed the association between iron supplementation and *P. vivax* malaria, there was an increase in risk of first episode of *P. vixax* parasitemia associated with iron supplementation, but only during the first 30 days (for 1–15 days aHR 1.75, 95% CI 1.14–2.70, p = 0.009) and 16–30 days (aHR 1.70, 95% CI 1.10–2.60, p = 0.01), after which the effect declined, and reversed for women who had taken iron supplementation for >60 days (aHR 0.67, 95% CI 0.40–1.02, p = 0.06, table 5) [38].

### Parenteral Iron Treatment and Malaria Risk

Associations between parenteral iron treatment among severely anemic pregnant women and malaria risk were evaluated in one retrospective and one prospective study (table 6) [62], [63]. The retrospective study compared malaria during the post-natal period between severely anemic pregnant women treated during pregnancy with a single intravenous dose of 1750 mg of iron to women without anemia. A marked increase in the odds of postnatal malaria was observed among primiparous women (OR 5.5, 95% CI 2.2–13.5) whereas this association was not seen among multiparious women (OR 1.1, 95% CI 0.73–1.70) [63]. The prospective cohort study assessed the incidence of local and general reactions to total dose infusion of iron dextran among four groups of pregnant women: A: iron only; B: iron+antihistamine; C: iron+chloroquine; D: iron+antihistamine+chloroquine. However, they only assessed the presence of malaria following infusion among those women with general reactions. Among women with general reactions, those not concurrently receiving chloroquine with their iron infusion were more likely to have malaria parasitemia after the infusion compared to women who did receive concurrent chloroquine (28.2% vs. 0%, respectively; p = 0.17) [62]. Indications, timing of iron treatment and malaria assessment between the studies were too dissimilar to perform a pooled analysis.

**Table 6:** Summary of study outcomes – Iron Treatment vs. Malaria Risk.

Author, Year	Study Design	Outcome	Exposure% (n)	Comparison to exposure% (n)	Risk estimate (95% CI)or p-value
Oppenheimer, 1986 [63]	Retrospective Cohort	Postnatal malaria- primigravida	Total Dose Iron Infusion 20·4% (11/54)	No Total Dose Iron Infusion 4·5% (3/67)	RR 4·55 (1·34–15·49) OR 5·46 (1·44, 20·7)
		Postnatal malaria- multigravida	Total Dose Iron Infusion 8·4% (8/95)	No Total Dose Iron Infusion 7·6% (14/184)	RR 1·11 (0·48–2·54) OR 1·12 (0·45, 2·76)
Byles, 1970 [62]	Prospective Cohort	Generalized reactions after total dose iron infusion	Total dose iron+Concurrent CQ 1·5% (7/462)	Total dose iron+No concurrent CQ 8·6% (39/455)	OR 0·16 (0·07–0·37) RR 0·18 (0·08–0·39)
		PF parasitemia present among participants with generalized reactions after total dose iron infusion	Total dose iron+Concurrent CQ 0·0% (0/7)	Total dose iron+No concurrent CQ 28·2% (11/39)	p = 0·17

CI: confidence interval. CQ: chloroquine. OR: odds ratio. PF: *Plasmodium falciparum*. RR: risk ratio.

### Iron Deficiency and Malaria Risk

**Figure 3:**
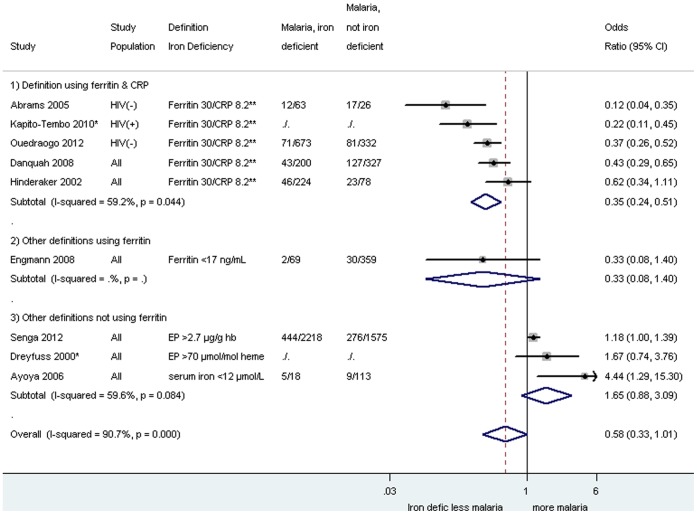
Malaria parasitemia by blood smear among iron deficient and non-deficient participants, by definition of iron deficiency during pregnancy. Footnote: CI: confidence interval. CRP: C-Reactive Protein. EP: Erthyrocyte protoporphyrin. Hb: hemoglobin. *Use of adjusted odds ratios: Kapito-Tembo 2010: odds ratio adjusted for CD4 count, gravidity, and intestinal infections, Dreyfuss: odds ratio adjusted for hookworm infection, serum retinol and trimester of pregnancy. **Iron deficiency definition: Ferritin <30 ng/mL & CRP< = 8.2 ng/mL or ferritin <70 ng/mL & CRP>8.2 ng/mL. The weight for each study is indicated as a grey block around the risk estimate. For Dreyfuss 2000, malaria parasitemia was limited to *P. vivax* in Asia. All other studies were conducted in Africa where *P. falciparum* is the predominant species.

**Figure 4:**
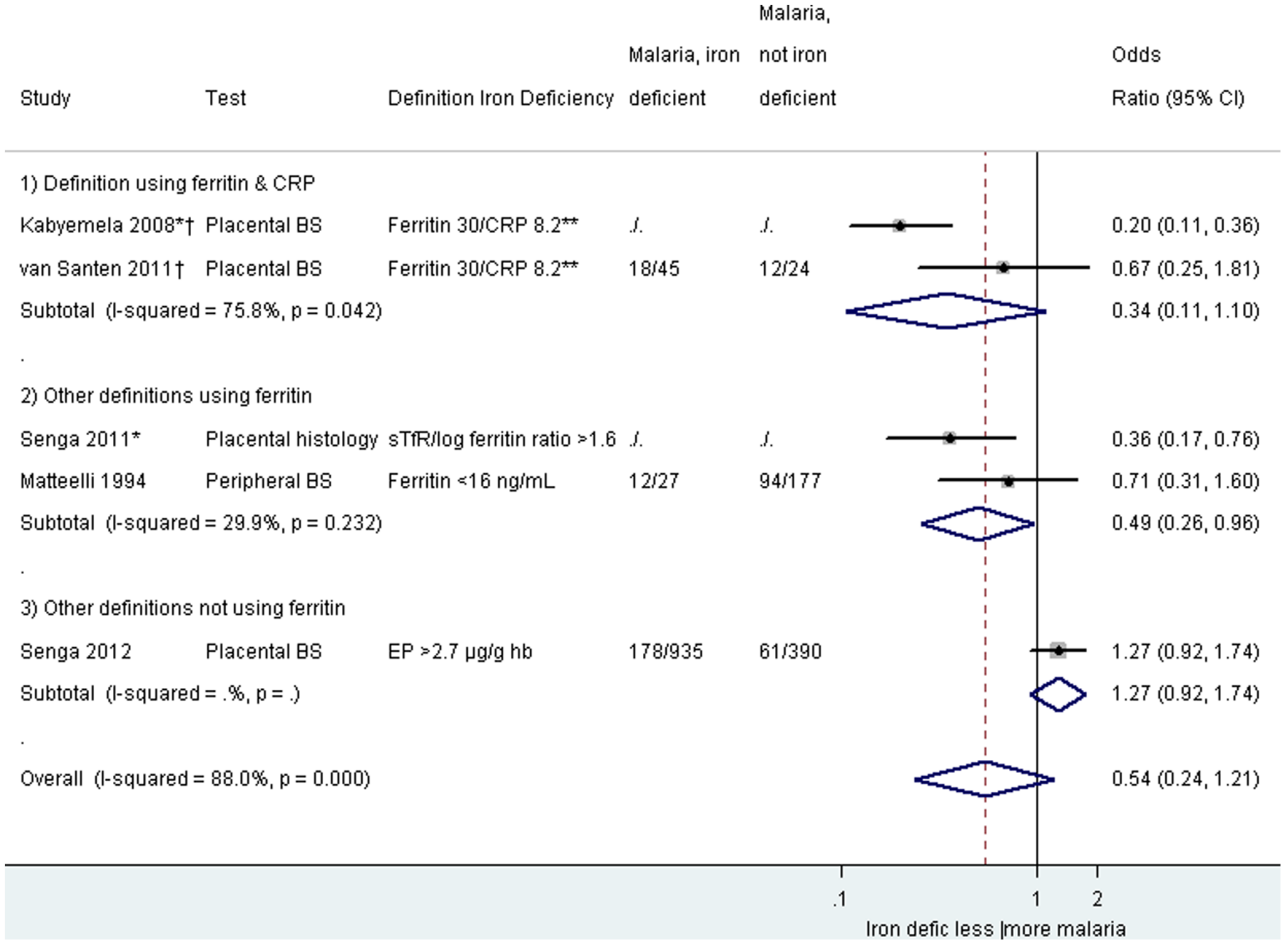
Malaria parasitemia among iron deficient and non-deficient participants, by definition of iron deficiency at the time of delivery. Footnote: BS: bloodsmear. CI: confidence interval. CRP: C-reactive protein. EP: Erthyrocyte protoporphyrin. Hb: hemoglobin. sTfR: Soluble transferrin receptor. *Adjusted odds ratios used: Kabyemela 2008: odds ratio adjusted for gravidity. Senga 2011: odds ratio adjusted for gravidity, age, and blood group. Placental histology in Senga 2011: active infection defined by acute or chronic infection (parasites alone, or parasites in the presence of haemozoin). No placental infection defined by the absence of parasites and haemozoin. ** Ferritin 30/CRP 8.3: Ferritin <30 ng/mL & CRP< = 8.2 ng/mL or ferritin <70 ng/mL & CRP>8.2 ng/mL. The weight for each study is indicated as a grey block around the risk estimate.

The effect of iron deficiency on malaria risk in pregnancy was assessed in 12 studies including three case-control studies [40], [47], [49], and nine cross-sectional studies (table 2) [34], [41]–[46], [48], [50]. Among these studies, five considered evidence of malaria at the time of delivery [40], [44], [48]–[50], six considered malaria at the time of enrollment [34], [41]–[43], [45], [47], and one did not specify when iron deficiency was assessed [46]. Seven of these 12 studies used the same definitions of iron deficiency (serum ferritin <30 ng/mL with CRP< = 8.2 ng/mL or serum ferritin <70 ng/mL with CRP>8.2 ng/mL), and pooled analysis showed an overall decreased odds for peripheral parasitemia in pregnancy (OR_pooled_ 0.35, 95% CI 0.24–0.51; I^2^ 59.2%, five studies, figure 3) associated with iron deficiency, but this was not significant for placental malaria (OR_pooled_ 0.34, 95% CI 0.11–1.10; I^2^ 75·8%, two studies, figure 4). Other definitions using ferritin tended to show similar results with decreased odds of malaria among iron-deficient women (figure 3 and 4); however, where definitions were used which did not include ferritin, no overall effect was seen (OR_pooled_ for malaria in pregnancy: 1.65, 95% CI 0.88–3.09, I^2^ 59.6%, three studies, figure 3). Additional iron deficiency definitions and malaria outcomes which could not be included because of overlap of studies are listed in the supplement File S1. A subgroup analysis was conducted by gravidity which indicated that the association between malaria parasitaemia and markers of iron status were similar among the different gravidae groups (Figure S3.2 in File S1).

### Iron Biomarkers and Malaria Risk

**Figure 5:**
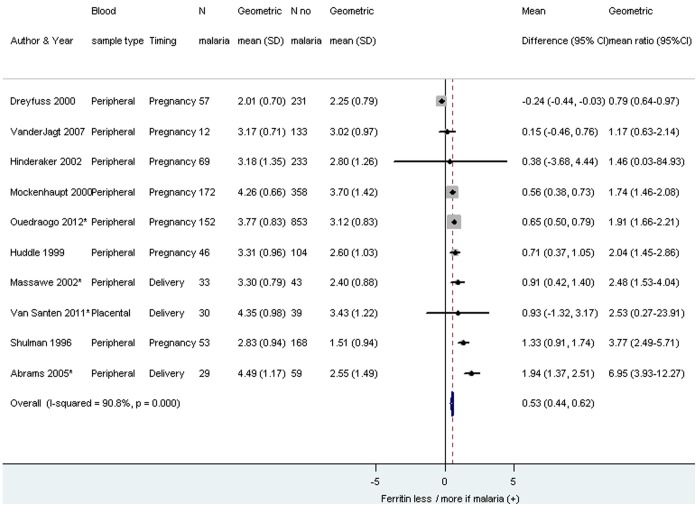
Geometric mean difference in ferritin among pregnant women infected with malaria compared to pregnant women without malaria. Footnote: CI: confidence interval. SD: standard deviation. *Van Santen 2011 and Massawe 2002: study population primigravidae. Abrams 2005 and Ouedraogo 2012: study population HIV(-) women. The weight for each study is indicated as a grey block around the risk estimate. For Dreyfuss 2000, the malaria parasitemia was limited to *P. Vivax* in Asia. All other studies were conducted in Africa where *P. falciparum* is the predominant species.

Iron biomarkers evaluated included serum ferritin, serum iron, total iron binding capacity, soluble transferrin receptor, transferrin saturation (as %) and serum transferrin (figure 4). Studies which could not be included in forest plots because of insufficient information are presented in Table 7. The pooled analysis of serum ferritin was conducted among ten studies for which geometric mean values were provided or could be calculated and showed that the concentration of serum ferritin was higher among malaria infected pregnant women compared to those who were uninfected (geometric mean difference_pooled_ 0.53, 95% CI 0.44–0.62, I^2^ 90.8%, figure 5), which corresponds to ferritin levels being 70% (95% CI 55–86%) higher in women with malaria compared to those without.

**Table 7:** Summary of study outcomes – Iron biomarkers vs. malaria risk, and outcomes not presented in forest plot.

Author, Year	Study Design	Biomarker(Outcome)	Exposure% (n or IQR)	Comparison to exposure% Outcome (n or IQR)	p-value
Engmann 2008 [41]	Cross-sectional	Serum ferritin (μg/L)	Peripheral parasitemia at enrollment Median 91 (IQR: 33, 157)	No peripheral parasitemia at enrollment Median 33 (IQR: 20, 50)	Not provided
Matteelli 1994 [42]	Cross-sectional	Serum ferritin (ng/mL)	Peripheral parasitemia at delivery Mean 65.7 (n = 106)	No peripheral parasitemia at delivery Mean 36.5 (n = 98)	p = 0.002
Ndyomugyenyi 2008 [61]	Cross-sectional	Serum ferritin (μg/L)	Peripheral parasitemia at enrollment (Means not available)	No peripheral parasitemia at enrollment (means not available)	p = 0.007
Saad 2012 [52]	Case-control	Serum ferritin (μg/L)	Peripheral parasitemia at enrollment (Uncomplicated malaria) Median 63.3 (IQR: 30.5, 113.2)	No peripheral parasitemia at enrollment Median 34.4 (IQR: 7.9, 60.3)	p = 0.041
		Serum ferritin (μg/L)	Peripheral parasitemia at enrollment (Severe malaria) Median 78.6 (IQR: 44.1, 148.9)	No peripheral parasitemia at enrollment Median 34.4 (IQR: 7.9, 60.3)	p = 0.002
Dreyfuss 2000 [46]	Cross-sectional	Erythrocyte Protoporhyrin (μmol/mol heme)	Peripheral parasitemia at enrollment Geometric mean 90 (CI 54, 150) (n = 57)	No peripheral parasitemia at enrollment Geometric mean 84 (CI 52, 134) (n = 231)	P = 0.3

CI: confidence interval. IQR: Interquartal range.

**Figure 6:**
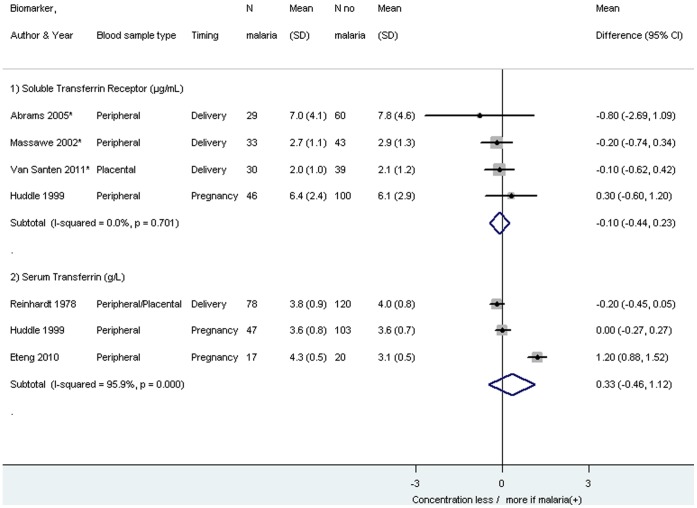
Mean difference in soluble transferring receptor and serum transferrin among pregnant women infected with malaria compared to pregnant women without malaria. Footnote: CI: confidence interval. SD: standard deviation. *Van Santen 2011 and Massawe 2002: study population primigravidae. Abrams 2005: study population HIV(-) women. The weight for each study is indicated as a grey block around the risk estimate. Removal of the study of Eteng 2010, an outlier for Serum Transferrin, gives the following result: Pooled mean difference Serum Transferrin (g/L): −0.11, 95% CI −0.30 to 0.09, I^2^ 14.8%.

**Figure 7:**
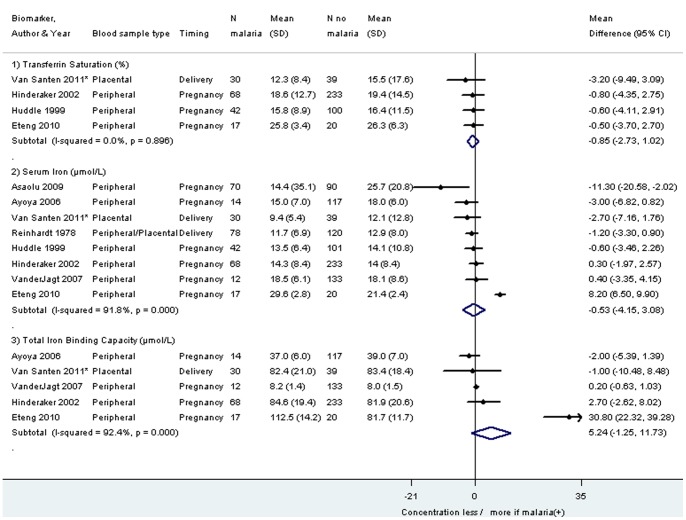
Mean difference in transferrin saturation, serum iron and total iron binding capacity among pregnant women infected with malaria compared to pregnant women without malaria. Footnote: CI: confidence interval. SD: standard deviation. *Van Santen 2011: study population primigravidae. The weight for each study is indicated as a grey block around the risk estimate. Removal of the study of Eteng 2010, an outlier for Serum Iron and Total Iron Binding Capacity, gives the following results: Pooled mean difference serum iron (μmol/L): −1.13, −2.57 to 0.31, I^2^ 27.3%; Pooled mean difference Total Iron Binding Capacity (μmol/L): 0.13, −0.67 to 0.92, I-squared 0%.

The same pattern was seen in four studies which could not be included in the meta-analysis (table 7). No significant pooled differences were detected for serum iron (eight studies), total iron binding capacity (five studies), soluble transferrin receptor (four studies), serum transferrin (three studies), and tranferrin saturation (four studies), whereby it should be noted that the heterogeneity was 0% for soluble transferrin receptor and transferrin saturation (figure 6 and 7).

## Discussion

To our knowledge, this systematic review represents the first effort to assess the association of iron status and use on the risk of malaria in pregnancy. Pooled analyses of iron deficiency status, using a definition based on ferritin and adjusting for inflammation with CRP, indicated pregnant women with iron deficiency had a reduced risk of malaria infection during pregnancy. However, pooled analyses from the remaining biomarkers of iron deficiency were not associated with malaria. Data from randomized and observational studies did not show an increased risk of *P. falciparum* malaria among the participants who received oral iron supplementation, whereas one cohort study in Asia showed an increased risk of *P. vivax* in the first 30 days of oral iron (and folate) supplementation. None of the studies however, assessed the individual’s baseline iron status which may modify the effect of iron supplementation on malaria risk, complicating the interpretation of these data. Except for the studies using parenteral iron treatment, all included studies used oral iron supplementation for the prevention of iron deficiency anemia during pregnancy; oral iron was not given for the treatment of established anemia and/or iron-deficiency in these studies. This difference can be important because the treatment of anemia in pregnancy recommends using temporarily higher doses of iron, specific policies for iron dose and duration vary by country [7].

Only two studies evaluated parenteral treatment with iron dextran and the study qualities were insufficient to make conclusions. In addition, new parenteral iron treatment options using complex iron-polysaccharides are available which may not have a similar risk profile with regards to infections such as malaria [64].

Several hypotheses have been provided as to why malaria infection may be less prevalent among people with iron deficiency. Iron is vital for the survival of the malaria parasite and the parasite is unable to thrive in iron-deficient environments [65]. Specifically, iron deficiency suppresses erythropoiesis, the production of red blood cells, thereby reducing the opportunity for the parasite to infect the host [66]. Additionally, some aspects of the host immunity against malaria may be boosted with iron deficiency. For example, the host macrophage defense utilizes nitric oxide in the control of *P. falciparum* and because iron can down-regulate the formation of nitric oxide within macrophages [67], iron deficiency may therefore enable a stronger host-macrophage response.

Evidence from iron chelation studies using desferrioxamine (DFO) provides support that iron-deficient environments are unfavorable to foster malaria infections. In one randomized double-blind placebo cross-over study, DFO among asymptomatic parasitemic adults was associated with enhanced parasite clearance compared to placebo [68]. Another double-blind randomized study among children with cerebral malaria showed the median recovery time from children in deep coma was reduced by half (and the rate of parasite clearance was improved) when DFO was added to treatment with quinine compared to the antimalarial treatment plus placebo [69].

Multiple iron biomarkers have been associated with malaria infection among various populations. The most widely reported is serum ferritin, a known acute-phase reactant protein that can increase in response to underlying infection or inflammation regardless of underlying iron stores [55], [70]–[76]. Our data confirm serum ferritin is increased in the presence of malaria infection in pregnant women. The reported association between malaria and iron deficiency reported above adjusted for the effect of inflammation. Results from our pooled analysis did not find statistically significant associations between the remaining biomarkers and malaria. These results are in agreement with findings in the literature among non-pregnant populations where associations have been inconsistent between malaria and: sTfR [21], [70], [72], [73], [77], [78], transferrin [72], [73], and transferrin saturation [72].

Despite the evidence that serum ferritin increases as a result of pregnancy per se, infection or inflammation, it remains the international standard for defining iron deficiency [79]. This creates an inherent challenge to measuring iron deficiency using cutoffs of serum ferritin in pregnant populations living in malaria endemic settings (see also Figure S3.1 in File S1). Consensus exists that serum ferritin should be measured with inflammatory markers such as C-reactive protein, a1-antichymotrypsin, or a1-acid glycoprotein; however, agreement has yet to be made on the best inflammatory markers and their corresponding cutoff values for defining iron deficiency in the presence of inflammation [80]–[83]. Other iron biomarkers have been proposed to replace serum ferritin including sTfR. While it has been suggested sTfR is unaffected by pregnancy or inflammation [84], evidence of how pregnancy affects concentrations of sTfR is lacking [79]. The extent to which sTfR is affected by pregnancy will determine the appropriateness of using sTfR as a biomarker for detecting iron deficiency in pregnancy, especially in malaria endemic settings.

While the evidence of iron supplementation related to malaria risk in pregnancy is not of strong quality, the available data suggest iron supplementation does not increase the risk of malaria. Among the seven studies included in this review, only two of them represented primary data analyses from randomized trials, both of which failed to see an association between iron supplementation in pregnancy and malaria risk. No routine medical malaria prevention was used in these trials among the women included in this review, and they were conducted before the wide-scale introduction of insecticide treated nets [36], [37]. The same applied to the study using a before-after design [39].

Prevalence of use of malaria prevention strategies varied greatly between the two surveys included for iron supplementation, and these showed the greatest variety in the association between iron supplementation and malaria [34], [35]. One survey reported less malaria among iron supplemented (HIV-infected) women (figure 2) [34]; however, it should be noted, that we transformed their data from odds ratios to risk ratios to be able to combine the results of the different studies [34]. Kapito-Tembo (2010) reported an odds ratio of 0.4, 95% CI 0.14–1.12, in multivariable analysis, comparing iron supplemented vs. not supplemented when adjusting for age, gravidity, bed net use, socio-economic status, antenatal visits, IPTp use or cotrimoxazole prophylaxis [34].

The higher risk of malaria among women that used iron for >30 days in the subgroup analysis (Figure S2.3 in File S1) could potentially be explained by the association with IPTp [34], [35]. Women who use iron for ≤30 days may be protected by the last dose of IPTp with SP if this was given at the visit when the iron supplementation commenced. SP is known to provide four to six weeks of post-treatment prophylaxis against malaria after which protective drug levels wane, placing women who continue using iron at risk until they receive the next dose of SP. In addition, some women may have initially been iron deficient, but with continued supplementation, have become iron replete, which may contribute to development of malaria [18], [85]. Among children it has been suggested that an individual’s baseline iron status may modify the risk of malaria associated with iron supplementation; e.g. among iron-deficient persons, iron supplementation may decrease anemia, whereas among iron-replete persons, supplementation may have no benefits for anemia reduction and only result in an increased risk for malaria [21]. However, none of the iron supplementation studies included baseline iron status, although they all showed either a decrease in anemia (RR_pooled_ 0.79, 95% CI 0.73–0.85, four studies, I^2^ 31.7%, Figure S5.1 in File S1), or an improvement in hemoglobin (mean increase 0.79 g/dl, 95% CI 0.64–0.95, three studies, I^2^ 15·6%, Figure S5.2 in File S1). While direct evidence of risk associated with iron supplementation is lacking, strong evidence exists that iron supplementation improves hemoglobin levels and decreases the risk of anemia and improves newborn iron stores [8], [9], [86].

This review is subject to several limitations, an examination of which may inform the design and conduct of future studies of iron supplementation during pregnancy in malaria endemic areas. First, the 31 studies identified were widely heterogeneous. Among the seven studies with data on iron supplementation, the cumulative dose of iron used and the timing and type of malaria testing varied as did the study design and methodology reducing comparability and preventing a pooled analysis across all available studies. Blood smears used to be the standard for detecting malaria whereas now rapid diagnostic malaria tests, placental histology, and polymerase chain reactions are increasingly used, with the latter two tests having a higher sensitivity in detecting malaria [87]. Furthermore, the Entomological Inoculation Rate *(*a measure of malaria transmission in an area) and the prevalence of malaria varied greatly across these studies, as did the use of malaria prevention strategies, and the dose and duration of iron supplementation (table 1, 2, 3, 4). The distribution of biomarkers was not always normally distributed, and approximations of geometric means were made for ferritin. Consistency on these methodological aspects would allow for more accurate synthesis of the body of literature. The majority of these data are based on cross-sectional studies which provide evidence of an association between these factors, but cannot confirm a causal association between iron deficiency (measured by serum ferritin) and malaria risk. In addition, the direction of this association between malaria and iron status is unclear, as iron status may affect the malaria risk, but malaria infection itself causes an inflammatory response as well as hemolysis and will affect these measures of iron status. Lastly, with the exception of three studies, these data represent the risk associated with *Plasmodium falciparum* infections in Africa [38], [46], [63], and the effect of iron on malaria risk may differ by species and regions.

### Directions for Future Research

While in children in malaria endemic areas there has been enough evidence of increased risk of morbidity associated with iron use to result in a more restricted policy in this population, surprisingly little is known of the risk-benefits in pregnancy and universal iron supplementation continues to be recommended during pregnancy. Because the relationship between malaria and iron status is difficult to disentangle, descriptive studies are not adequate, and randomized placebo controlled intervention trials may be the only way to answer the causal relationship between iron and malaria infection. However, these are costly and there may be ethical considerations due the need to withhold an intervention that is policy in most countries and the proven health benefit of iron supplementation in pregnant women. Using a clinical trial register, we identified five studies in various stages which will start to address some of these issues; baseline assessment of iron status will be important as well as the use of different definitions of iron deficiency, preferably not all ferritin-based [88]–[92].

These data suggest that iron supplementation may be safe in malaria endemic areas among women concurrently using malaria prevention strategies such as insecticide treated nets, monthly IPTp or daily cotrimoxazol (in HIV-infected women). Although IPTp and insecticide treated nets are currently the main policies to prevent malaria in pregnant women in sub-Saharan Africa, their coverage is less than optimal and it would be important to ensure that individual women receiving iron supplementation are indeed protected by an insecticide treated net and receive IPTp [93]. Because IPTp with SP is contraindicated in the first trimester, it is important to ensure that women using iron early in pregnancy are using an insecticide treated net and are screened for malaria at each scheduled visit until they can receive IPTp. With some countries moving towards iron fortification, it will also be prudent to understand the potential risk and benefits of additional exposure to oral iron supplementation that is likely to be continued to be provided for some time, as part of routine antenatal care.

## Conclusion

Our review suggests iron supplementation may be safe during pregnancy in malaria endemic areas. However, the available data on iron supplementation in pregnancy and malaria risk are limited and insufficient to rule out any potential for an increased risk of malaria in malaria endemic settings. While iron deficiency (based on a definition with ferritin and CRP) is associated with a marked decreased risk of malaria in pregnancy, this association was not present among any of the other biomarkers of iron deficiency. Therefore, we find the results of iron deficiency and malaria risk to be inconclusive. Until more data are available from pregnant women, it would be prudent, based on the available evidence among children, to recommend that the provision of universal iron supplementation in pregnant women should always occur in conjunction with malaria prevention strategies during pregnancy in malaria endemic areas.

## Supporting Information

File S1 Supporting Information
(DOCX)

Checklist S1 PRISMA Checklist
(DOCX)

## References

[1] Gilles HM, Williams EJ, Ball PA (1964) Hookworm Infection and Anaemia. An Epidemiological, Clinical, and Laboratory Study. Q J Med 33: 1–24. 14116854

[2] Watson-Jones D, Weiss HA, Changalucha JM, Todd J, Gumodoka B, et al (2007) Adverse birth outcomes in United Republic of Tanzania–impact and prevention of maternal risk factors. Bull World Health Organ 85: 9–18. 17242753 10.2471/BLT.06.033258 PMC2636214

[3] Rush D (2000) Nutrition and maternal mortality in the developing world. Am J Clin Nutr 72: 212S–240S. 10871588 10.1093/ajcn/72.1.212S

[4] Gillespie S (1998) Major issues in the control of iron deficiency. New York, USA: The Micronutrient Initiative.

[5] Bundy DA, de Silva NR (1998) Can we deworm this wormy world? Br Med Bull 54: 421–432. 9830207 10.1093/oxfordjournals.bmb.a011698

[6] Klemm R, Sommerfelt AE, Boyo A, Barba C, Kotecha P, et al.. (2011) Are We Making Progress on Reducing Anemia in Women? Cross-country Comparison of Anemia Prevalence, Reach, and Use of Antenatal Care and Anemia Reduction Interventions. Washington DC, USA: A2Z: The USAID Micronutriend and Child Blindness Project.

[7] Stoltzfus R, Dreyfuss M (1998) Guidelines for the use of iron supplements to prevent and treat iron deficiency anaemia. ILSI Press, Washington, DC.

[8] Pena-Rosas JP, De-Regil LM, Dowswell T, Viteri FE (2012) Daily oral iron supplementation during pregnancy. Cochrane Database Syst Rev 12: CD004736. 23235616 10.1002/14651858.CD004736.pub4 PMC4233117

[9] Rao R, Georgieff MK (2001) Neonatal iron nutrition. Semin Neonatol 6: 425–435. 11988032 10.1053/siny.2001.0063

[10] Hay G, Sandstad B, Whitelaw A, Borch-Iohnsen B (2004) Iron status in a group of Norwegian children aged 6–24 months. Acta Paediatr 93: 592–598. 15174778

[11] Georgieff MK, Wewerka SW, Nelson CA, Deregnier RA (2002) Iron status at 9 months of infants with low iron stores at birth. J Pediatr 141: 405–409. 12219063 10.1067/mpd.2002.127090

[12] Lozoff B (2007) Iron deficiency and child development. Food Nutr Bull 28: S560–571. 18297894 10.1177/15648265070284S409

[13] Lozoff B, Georgieff MK (2006) Iron deficiency and brain development. Semin Pediatr Neurol 13: 158–165. 17101454 10.1016/j.spen.2006.08.004

[14] Titaley CR, Dibley MJ, Roberts CL, Agho K (2010) Combined iron/folic acid supplements and malaria prophylaxis reduce neonatal mortality in 19 sub-Saharan African countries. Am J Clin Nutr 92: 235–243. 20504976 10.3945/ajcn.2009.29093

[15] Eisele TP, Larsen DA, Anglewicz PA, Keating J, Yukich J, et al (2012) Malaria prevention in pregnancy, birthweight, and neonatal mortality: a meta-analysis of 32 national cross-sectional datasets in Africa. Lancet Infect Dis 12: 942–949. 22995852 10.1016/S1473-3099(12)70222-0

[16] Oppenheimer SJ, Gibson FD, Macfarlane SB, Moody JB, Harrison C, et al (1986) Iron supplementation increases prevalence and effects of malaria: report on clinical studies in Papua New Guinea. Trans R Soc Trop Med Hyg 80: 603–612. 3101243 10.1016/0035-9203(86)90154-9

[17] Gwamaka M, Kurtis JD, Sorensen BE, Holte S, Morrison R, et al (2012) Iron Deficiency Protects Against Severe Plasmodium falciparum Malaria and Death in Young Children. Clin Infect Dis 54: 1137–1144. 22354919 10.1093/cid/cis010 PMC3309886

[18] Prentice AM, Ghattas H, Doherty C, Cox SE (2007) Iron metabolism and malaria. Food Nutr Bull 28: S524–539. 18297891 10.1177/15648265070284S406

[19] Sazawal S, Black RE, Ramsan M, Chwaya HM, Stoltzfus RJ, et al (2006) Effects of routine prophylactic supplementation with iron and folic acid on admission to hospital and mortality in preschool children in a high malaria transmission setting: community-based, randomised, placebo-controlled trial. Lancet 367: 133–143. 16413877 10.1016/S0140-6736(06)67962-2

[20] Ojukwu JU, Okebe JU, Yahav D, Paul M (2009) Oral iron supplementation for preventing or treating anaemia among children in malaria-endemic areas. Cochrane Database Syst Rev: CD006589. 10.1002/14651858.CD006589.pub2 19588399

[21] Raiten D, Namaste S, Brabin B (2012) Considerations for the safe and effective use of iron interventions in areas of malaria burden. Bethesda, USA: NICHD, NIH, HHS. 10.1024/0300-9831/a000051 22002219

[22] Malaria in Pregnancy Consortium Malaria in Pregnancy Library. Available: http://library.mip-consortium.org. Accessed 27 November 2013.

[23] Liberati A, Altman DG, Tetzlaff J, Mulrow C, Gotzsche PC, et al (2009) The PRISMA statement for reporting systematic reviews and meta-analyses of studies that evaluate health care interventions: explanation and elaboration. PLoS Med 6: e1000100. 19621070 10.1371/journal.pmed.1000100 PMC2707010

[24] Higgins JP, Altman DG, Gotzsche PC, Juni P, Moher D, et al (2011) The Cochrane Collaboration’s tool for assessing risk of bias in randomised trials. BMJ 343: d5928. 22008217 10.1136/bmj.d5928 PMC3196245

[25] Sanderson S, Tatt ID, Higgins JP (2007) Tools for assessing quality and susceptibility to bias in observational studies in epidemiology: a systematic review and annotated bibliography. Int J Epidemiol 36: 666–676. 17470488 10.1093/ije/dym018

[26] von Elm E, Altman DG, Egger M, Pocock SJ, Gotzsche PC, et al (2007) The Strengthening the Reporting of Observational Studies in Epidemiology (STROBE) statement: guidelines for reporting observational studies. Bull World Health Organ 85: 867–872. 18038077 10.2471/BLT.07.045120 PMC2636253

[27] Banjoko SO, Oseni FA, Togun RA, Onayemi O, Emma-Okon BO, et al (2012) Iron status in HIV-1 infection: implications in disease pathology. BMC Clin Pathol 12: 26. 23245266 10.1186/1472-6890-12-26 PMC3551638

[28] Finkelstein JL, Mehta S, Duggan CP, Spiegelman D, Aboud S, et al (2012) Predictors of anaemia and iron deficiency in HIV-infected pregnant women in Tanzania: a potential role for vitamin D and parasitic infections. Public Health Nutr 15: 928–937. 22014374 10.1017/S1368980011002369 PMC3366262

[29] Higgins JP, White IR, Anzures-Cabrera J (2008) Meta-analysis of skewed data: combining results reported on log-transformed or raw scales. Stat Med 27: 6072–6092. 18800342 10.1002/sim.3427 PMC2978323

[30] Friedrich JO, Adhikari NK, Beyene J (2012) Ratio of geometric means to analyze continuous outcomes in meta-analysis: comparison to mean differences and ratio of arithmetic means using empiric data and simulation. Stat Med 31: 1857–1886. 22438170 10.1002/sim.4501

[31] DerSimonian R, Kacker R (2007) Random-effects model for meta-analysis of clinical trials: an update. Contemp Clin Trials 28: 105–114. 16807131 10.1016/j.cct.2006.04.004

[32] Higgins JP, Thompson SG, Deeks JJ, Altman DG (2003) Measuring inconsistency in meta-analyses. BMJ 327: 557–560. 12958120 10.1136/bmj.327.7414.557 PMC192859

[33] Menendez C, Todd J, Alonso PL, Francis N, Lulat S, et al (1995) The response to iron supplementation of pregnant women with the haemoglobin genotype AA or AS. Trans R Soc Trop Med Hyg 89: 289–292. 7660438 10.1016/0035-9203(95)90546-4

[34] Kapito-Tembo A (2010) Malaria and anemia in HIV-infected pregnant women in Malawi: Associations with cotrimoxazole prophylaxis, submicroscopic malaria, iron supplementation and iron deficiency: Thesis. University of North Carolina at Chapel Hill. 135 p.

[35] Mwapasa V (2004) The interactions between Plasmodium Falciparum malaria and HIV-1 in pregnant Malawian women: Thesis. The University of Michigan. 155 p.

[36] Ndyomugyenyi R, Magnussen P (2000) Chloroquine prophylaxis, iron/folic-acid supplementation or case management of malaria attacks in primigravidae in western Uganda: effects on congenital malaria and infant haemoglobin concentrations. Ann Trop Med Parasitol 94: 759–768 discussion 769–770. 11214094 10.1080/00034980020015189

[37] Menendez C, Todd J, Alonso PL, Francis N, Lulat S, et al (1994) The effects of iron supplementation during pregnancy, given by traditional birth attendants, on the prevalence of anaemia and malaria. Trans R Soc Trop Med Hyg 88: 590–593. 7992349 10.1016/0035-9203(94)90176-7

[38] Nacher M, McGready R, Stepniewska K, Cho T, Looareesuwan S, et al (2003) Haematinic treatment of anaemia increases the risk of Plasmodium vivax malaria in pregnancy. Trans R Soc Trop Med Hyg 97: 273–276. 15228240 10.1016/s0035-9203(03)90140-4

[39] van Eijk AM, Ayisi JG, Slutsker L, Ter Kuile FO, Rosen DH, et al (2007) Effect of haematinic supplementation and malaria prevention on maternal anaemia and malaria in western Kenya. Trop Med Int Health 12: 342–352. 17313505 10.1111/j.1365-3156.2006.01787.x

[40] Abrams ET, Kwiek JJ, Mwapasa V, Kamwendo DD, Tadesse E, et al.. (2005) Malaria during pregnancy and foetal haematological status in Blantyre, Malawi. Malar J 4. 10.1186/1475-2875-4-39 PMC1232864 16122391

[41] Engmann C, Adanu R, Lu TS, Bose C, Lozoff B (2008) Anemia and iron deficiency in pregnant Ghanaian women from urban areas. Int J Gynaecol Obstet 101: 62–66. 18068171 10.1016/j.ijgo.2007.09.032

[42] Matteelli A, Donato F, Shein A, Muchi JA, Leopardi O, et al (1994) Malaria and anaemia in pregnant women in urban Zanzibar, Tanzania. Ann Trop Med Parasitol 88: 475–483. 7979637 10.1080/00034983.1994.11812894

[43] Ouedraogo S, Koura GK, Accrombessi MM, Bodeau-Livinec F, Massougbodji A, et al (2012) Maternal anemia at first antenatal visit: prevalence and risk factors in a malaria-endemic area in Benin. Am J Trop Med Hyg 87: 418–424. 22826498 10.4269/ajtmh.2012.11-0706 PMC3435342

[44] Senga EL, Koshy G, Brabin BJ (2012) Zinc erythrocyte protoporphyrin as marker of malaria risk in pregnancy - a retrospective cross-sectional and longitudinal study. Malar J 11: 249. 22846214 10.1186/1475-2875-11-249 PMC3464799

[45] Danquah I, Bedu-Addo G, Mockenhaupt FP (2008) Iron deficiency and Plasmodium falciparum infection during pregnancy. J Infect Dis 198: 1573–1574. 18956987 10.1086/592449

[46] Dreyfuss ML, Stoltzfus RJ, Shrestha JB, Pradhan EK, LeClerq SC, et al (2000) Hookworms, malaria and vitamin A deficiency contribute to anemia and iron deficiency among pregnant women in the plains of Nepal. J Nutr 130: 2527–2536. 11015485 10.1093/jn/130.10.2527

[47] Hinderaker SG, Olsen BE, Lie RT, Bergsjo PB, Gasheka P, et al (2002) Anemia in pregnancy in rural Tanzania: associations with micronutrients status and infections. Eur J Clin Nutr 56: 192–199. 11960293 10.1038/sj.ejcn.1601300

[48] Kabyemela ER, Fried M, Kurtis JD, Mutabingwa TK, Duffy PE (2008) Decreased susceptibility to Plasmodium falciparum infection in pregnant women with iron deficiency. J Infect Dis 198: 163–166. 18500927 10.1086/589512

[49] Senga EL, Harper G, Koshy G, Kazembe PN, Brabin BJ (2011) Reduced risk for placental malaria in iron deficient women. Malar J 10: 47. 21345193 10.1186/1475-2875-10-47 PMC3050778

[50] Van Santen S, de Mast Q, Luty AJ, Wiegerinck ET, Van der Ven AJ, et al (2011) Iron homeostasis in mother and child during placental malaria infection. Am J Trop Med Hyg 84: 148–151. 21212218 10.4269/ajtmh.2011.10-0250 PMC3005511

[51] Ayoya MA, Spiekermann-Brouwer GM, Traore AK, Stoltzfus RJ, Garza C (2006) Determinants of anemia among pregnant women in Mali. Food Nutr Bull 27: 3–11. 16572713 10.1177/156482650602700101

[52] Saad AA, Mohamed OE, Ali AA, Bashir AM, Ali NI, et al (2012) Acute-phase proteins in pregnant Sudanese women with severe Plasmodium falciparum malaria. Trans R Soc Trop Med Hyg 106: 570–572. 22818740 10.1016/j.trstmh.2012.06.004

[53] Asalou MF, Igbaakin PA (2009) Serum Levels of micronutrients and antioxidants during malaria in pregnant women In Ado-Ekiti, Ekiti State, Nigeria. International Journal of Medicine and Medical Sciences 1: 523–526.

[54] Eteng MU, Ekwe AO, Eyong EU, Ibekwe HA, Abolaji AO, et al (2010) Biochemical and haematological changes in pregnant malaria patients and pregnant non-malaria women. Scientific Research and Essays 59: 1009–1013.

[55] Huddle JM, Gibson RS, Cullinan TR (1999) The impact of malarial infection and diet on the anaemia status of rural pregnant Malawian women. Eur J Clin Nutr 53: 792–801. 10556986 10.1038/sj.ejcn.1600851

[56] Massawe SN, Ronquist G, Nystrom L, Lindmark G (2002) Iron status and iron deficiency anaemia in adolescents in a Tanzanian suburban area. Gynecol Obstet Invest 54: 137–144. 12571434 10.1159/000067879

[57] Mockenhaupt FP, Rong B, Gunther M, Beck S, Till H, et al (2000) Anaemia in pregnant Ghanaian women: importance of malaria, iron deficiency, and haemoglobinopathies. Trans R Soc Trop Med Hyg 94: 477–483. 11132370 10.1016/s0035-9203(00)90057-9

[58] Reinhardt MC (1978) Maternal anaemia in Abidjan–Its influence on placenta and newborns. Helv Paediatr Acta Suppl: 43–63. 215575

[59] Shulman CE, Graham WJ, Jilo H, Lowe BS, New L, et al (1996) Malaria is an important cause of anaemia in primigravidae: evidence from a district hospital in coastal Kenya. Trans R Soc Trop Med Hyg 90: 535–539. 8944266 10.1016/s0035-9203(96)90312-0

[60] VanderJagt DJ, Brock HS, Melah GS, El-Nafaty AU, Crossey MJ, et al (2007) Nutritional factors associated with anaemia in pregnant women in northern Nigeria. J Health Popul Nutr 25: 75–81. 17615906 PMC3013266

[61] Ndyomugyenyi R, Kabatereine N, Olsen A, Magnussen P (2008) Malaria and hookworm infections in relation to haemoglobin and serum ferritin levels in pregnancy in Masindi district, western Uganda. Trans R Soc Trop Med Hyg 102: 130–136. 17996912 10.1016/j.trstmh.2007.09.015

[62] Byles AB, D’sa A (1970) Reduction of reaction due to iron dextran infusion using chloroquine. Br Med J 3: 625–627. 4919019 10.1136/bmj.3.5723.625 PMC1701730

[63] Oppenheimer SJ, Macfarlane SB, Moody JB, Harrison C (1986) Total dose iron infusion, malaria and pregnancy in Papua New Guinea. Trans R Soc Trop Med Hyg 80: 818–822. 3603623 10.1016/0035-9203(86)90393-7

[64] Silverstein SB, Rodgers GM (2004) Parenteral iron therapy options. Am J Hematol 76: 74–78. 15114602 10.1002/ajh.20056

[65] Oppenheimer SJ (2001) Iron and its relation to immunity and infectious disease. J Nutr 131: 616S–633S discussion 633S–635S. 11160594 10.1093/jn/131.2.616S

[66] Li H, Ginzburg YZ (2010) Crosstalk between Iron Metabolism and Erythropoiesis. Adv Hematol 2010: 605435. 20631898 10.1155/2010/605435 PMC2902017

[67] Weiss G, Werner-Felmayer G, Werner ER, Grunewald K, Wachter H, et al (1994) Iron regulates nitric oxide synthase activity by controlling nuclear transcription. J Exp Med 180: 969–976. 7520477 10.1084/jem.180.3.969 PMC2191642

[68] Gordeuk VR, Thuma PE, Brittenham GM, Zulu S, Simwanza G, et al (1992) Iron chelation with desferrioxamine B in adults with asymptomatic Plasmodium falciparum parasitemia. Blood 79: 308–312. 1730079

[69] Gordeuk V, Thuma P, Brittenham G, McLaren C, Parry D, et al (1992) Effect of iron chelation therapy on recovery from deep coma in children with cerebral malaria. N Engl J Med 327: 1473–1477. 1406879 10.1056/NEJM199211193272101

[70] Asobayire FS, Adou P, Davidsson L, Cook JD, Hurrell RF (2001) Prevalence of iron deficiency with and without concurrent anemia in population groups with high prevalences of malaria and other infections: a study in Cote d’Ivoire. Am J Clin Nutr 74: 776–782. 11722959 10.1093/ajcn/74.6.776

[71] Cook JD, Skikne BS, Bayne RD (1996) The use of the serum transferrin receptor for the assessment of iron status. In: Iron Nutrition in Health and Disease, L Hallberg & N-G Asp ed., London: John Libbey & Co., 49–58.

[72] Das BS, Thurnham DI, Das DB (1997) Influence of malaria on markers of iron status in children: implications for interpreting iron status in malaria-endemic communities. Br J Nutr 78: 751–760. 9389898 10.1079/bjn19970192

[73] Menendez C, Quinto LL, Kahigwa E, Alvarez L, Fernandez R, et al (2001) Effect of malaria on soluble transferrin receptor levels in Tanzanian infants. Am J Trop Med Hyg 65: 138–142. 11508389 10.4269/ajtmh.2001.65.138

[74] Stoltzfus RJ, Chwaya HM, Albonico M, Schulze KJ, Savioli L, et al (1997) Serum ferritin, erythrocyte protoporphyrin and hemoglobin are valid indicators of iron status of school children in a malaria-holoendemic population. J Nutr 127: 293–298. 9039830 10.1093/jn/127.2.293

[75] Thurnham DI, McCabe GP, Northrop-Clewes CA, Nestel P (2003) Effects of subclinical infection on plasma retinol concentrations and assessment of prevalence of vitamin A deficiency: meta-analysis. Lancet 362: 2052–2058. 14697804 10.1016/s0140-6736(03)15099-4

[76] Verhoef H, West CE, Ndeto P, Burema J, Beguin Y, et al (2001) Serum transferrin receptor concentration indicates increased erythropoiesis in Kenyan children with asymptomatic malaria. Am J Clin Nutr 74: 767–775. 11722958 10.1093/ajcn/74.6.767

[77] Kuvibidila S, Mark JA, Warrier RP, Yu L, Ode D, et al (1995) Soluble transferrin receptor as an index of iron status in Zairian children with malaria. J Trop Med Hyg 98: 373–378. 8544218

[78] Williams TN, Maitland K, Rees DC, Peto TE, Bowden DK, et al (1999) Reduced soluble transferrin receptor concentrations in acute malaria in Vanuatu. Am J Trop Med Hyg 60: 875–878. 10344669 10.4269/ajtmh.1999.60.875

[79] Wheeler S (2008) Assessment and interpretation of micronutrient status during pregnancy. Proc Nutr Soc 67: 437–450. 18847521 10.1017/S0029665108008732

[80] Cook JD (2005) Diagnosis and management of iron-deficiency anaemia. Best Pract Res Clin Haematol 18: 319–332. 15737893 10.1016/j.beha.2004.08.022

[81] Raiten DJ, Namaste S, Brabin B, Combs G Jr, L’Abbe MR, et al (2011) Executive summary–Biomarkers of Nutrition for Development: Building a Consensus. Am J Clin Nutr 94: 633S–650S. 21733880 10.3945/ajcn.110.008227 PMC3142731

[82] World Health Organization (2011) Serum ferritin concentrations for the assessment of iron status and iron deficiency in populations. Vitamin and Mineral Nutrition Information System. Geneva, Switzerland.

[83] Zimmermann MB (2008) Methods to assess iron and iodine status. Br J Nutr 99 Suppl 3S2–9. 10.1017/S000711450800679X 18598585

[84] Carriaga MT, Skikne BS, Finley B, Cutler B, Cook JD (1991) Serum transferrin receptor for the detection of iron deficiency in pregnancy. Am J Clin Nutr 54: 1077–1081. 1957824 10.1093/ajcn/54.6.1077

[85] Clark M, Fisher NC, Kasthuri R, Cerami Hand C (2013) Parasite maturation and host serum iron influence the labile iron pool of erythrocyte stage Plasmodium falciparum. Br J Haematol 161: 262–269. 23398516 10.1111/bjh.12234 PMC4249674

[86] Pena-Rosas JP, Viteri FE (2009) Effects and safety of preventive oral iron or iron+folic acid supplementation for women during pregnancy. Cochrane Database Syst Rev: CD004736. 10.1002/14651858.CD004736.pub3 19821332

[87] Kattenberg JH, Ochodo EA, Boer KR, Schallig HD, Mens PF, et al (2011) Systematic review and meta-analysis: rapid diagnostic tests versus placental histology, microscopy and PCR for malaria in pregnant women. Malar J 10: 321. 22035448 10.1186/1475-2875-10-321 PMC3228868

[88] Gies S (2010) Long-term iron supplements and malaria risk in early pregnancy: a randomized controlled trial Available: http://clinicaltrials.gov/ct2/show/NCT01210040. Accessed 27 November 2013.

[89] Fawzi WW (2010) Trial of pre-pregnancy supplements. Available: http://clinicaltrials.gov/ct2/show/NCT01183572. Accessed 27 November 2013.

[90] Fawzi WW (2010) Prenatal iron supplements: safety and efficacy in Tanzania Available: http://clinicaltrials.gov/ct2/show/NCT01119612.Accessed 27 November 2013.

[91] Ashorn P (2010) Supplementing maternal and infant diet with high-energy, micronutrient fortified lipid-based nutrient supplements. Available: http://clinicaltrials.gov/ct2/show/NCT01239693. Accessed 27 November 2013.

[92] Verhoef H (2011) Prenatal iron and malaria study. Available: http://clinicaltrials.gov/ct2/show/NCT01308112. Accessed 27 November 2013.

[93] van Eijk AM, Hill J, Alegana VA, Kirui V, Gething PW, et al (2011) Coverage of malaria protection in pregnant women in sub-Saharan Africa: a synthesis and analysis of national survey data. Lancet Infect Dis 11: 190–207. 21273130 10.1016/S1473-3099(10)70295-4 PMC3119932

