# Supplementary material for: The Association between Malaria and Iron Status or Supplementation in Pregnancy: A Systematic Review and Meta-Analysis
Source: PLoS One. 2014 Feb 14;9(2):e87743. doi: 10.1371/journal.pone.0087743 (PMC3925104; doi:10.1371/journal.pone.0087743)
Supplement: File S1 — Supporting Information (DOCX) [file pone.0087743.s001.docx]

**Supporting Information for “Malaria and iron status or supplementation in pregnancy: a systematic review and meta-analysis”**

**Table of Contents**

1. [***Supporting information section 1***](#esup1)**: Quality assessment of studies included in “Malaria and iron status or supplementation in pregnancy”**
   1. [***Figure S1.1***](#efig11)**: Quality assessment of randomized clinical trials assessing effect of iron supplementation on malaria in pregnancy**
   2. [***Table S1.1***](#etab11)**: Randomized clinical trials quality assessment description**
   3. [***Figure S1.2***](#efig12)**: Quality assessment of observational studies (in alphabetical order)**
   4. [***Table S1.2***](#etab12)**: Observational studies quality assessment description**
2. [***Supporting information section 2***](#esup2)**: Supplementary graphs on malaria and iron supplementation in pregnancy**
   1. [***Figure S2.1***](#efig21): **Sub-group analysis malaria among iron-supplemented and non-supplemented pregnant women: HIV-status**
   2. [***Figure S2.2***](#efig22): **Sub-group analysis malaria among iron-supplemented and non-supplemented pregnant women: Sickle cell trait**
   3. [***Figure S2.3***](#efig23)**: Sub-group analysis malaria among iron-supplemented and non-supplemented pregnant women: duration of iron supplementation**
3. [***Supporting information section 3***](#esup3)**: Supplementary graphs on malaria and iron deficiency in pregnancy**
   1. [***Figure S3.1***](#efig31)**: Listing of additional definitions used for iron deficiency, and malaria risk among iron deficient and not-iron deficient women, sub-Saharan Africa and Asia**
   2. [***Figure S3.2***](#efig32): **Subgroup analysis by gravidity for malaria risk among iron deficient and not-iron deficient women, sub-Saharan Africa**
4. [***Supporting information section 4***](#esup4)**: Supplementary data on iron supplementation, iron status and malaria risk in pregnancy which has not been presented in forest plots**
   1. [***Table S4.1***](#etab51)**: Iron Supplementation vs. Malaria Risk in pregnancy: subgroup analysis (HIV) and less common malaria tests (PCR, placental histology)**
   2. [***Table S4.2***](#etab52)**: Iron Deficiency vs. Malaria Risk, sub group analysis (gravidity) and less common malaria tests (PCR)**
5. [***Supporting information section 5***](#esup5)**: Effect of iron supplementation on hemoglobin or anemia in pregnancy in iron supplementation studies included in this review**
   1. [***Figure S5*.*1***](#efig41)**: Effect of iron supplementation on hemoglobin level in studies included in this review**
   2. [***Figure S5.2***](#efig42): **Effect of iron supplementation on anemia** in **studies included in this review**
6. [***Supporting information references***](#ref)

***Supporting information section 1*: Quality assessment of studies included in “Malaria and iron status or supplementation in pregnancy”**

We used the Cochrane Collaboration’s tool for assessing the risk of bias [[1](#_ENREF_1)]. Assessment of the non-randomized study designs was based on source population, participant selection, completeness of exposure and outcome data, sample size, and measures to control confounding, after reviewing literature on quality assessment of observational studies [[2](#_ENREF_2),[3](#_ENREF_3)]. Scores were not assigned, but quality was assigned as low-to-moderate or good. An observational study was deemed to be good if there was an appropriate source population and participant selection, appropriate tests, and no substantial incomplete exposure or outcome data. Follow-up or outcome was considered adequate if more than 80% of participants initially enrolled were included in the analysis (and any loss was balanced across groups for trials), unclear if the percentage of initially enrolled participants included in the analysis was unclear, and inadequate if less than 80% of those initially enrolled were included in the analysis (or if loss was imbalanced in different treatment groups for trials).

***Figure S1*.*1*: Quality assessment of randomized clinical trials assessing effect of iron supplementation on malaria in pregnancy**

|  | **Random sequence generation (selection bias)** | **Allocation concealment (selection bias)** | **Blinding of participants and personnel (performance bias)** | **Blinding of outcome assessment (detection bias)** | **Incomplete outcome data (attrition bias)** | **Selective reporting (reporting bias)** | **Other bias** | **Final assessment quality trial** | **Notes** |
| --- | --- | --- | --- | --- | --- | --- | --- | --- | --- |
| Menendez 1994, 1995 [[4](#_ENREF_4),[5](#_ENREF_5)] | 🞇 | ▲ | ⬣ | ⬣ | ▲ | ▲ | ⬣ | Low | Sub group: multigravidae |
| Ndyomugyenyi 2000 [[6](#_ENREF_6)] | 🞇 | 🞇 | ⬣ | ⬣ | ▲ | ▲ | ⬣ | Low | Sub group: primigravidae |

⬣: Adequate or appropriate. 🞇: Unclear from available information. ▲: Inappropriate or inadaequate.

***Table S1.1*: Randomized clinical trials quality assessment description**

|  | **Random sequence generation** | **Allocation concealment** | **Blinding** | **Incomplete outcome data** | **Selective reporting** | **Other bias** |
| --- | --- | --- | --- | --- | --- | --- |
| **Menendez 1994, 1995 [**[**4**](#_ENREF_4)**,**[**5**](#_ENREF_5)**]** | “Allocated at random by compound or residence”  Unclear risk, method unclear | Not described. Active treatment and placebo were different colors.  High risk | Placebo used but different color than iron tablets.  Outcome assessor for malaria not aware of color of tablet. Not clear if e.g. study-code revealed treatment arm. Low risk. | High risk: more than 20% loss to follow up | Haematocrit <25% at follow up excluded from study (more in placebo arm than in treatment arm) | No other obvious bias  Sub group: multigravidae |
| **Ndyomugyenyi 2000 [**[**6**](#_ENREF_6)**]** | “Randomly assigned”  Unclear risk, method unclear | Not described. All arms used placebos (either CQ or iron/folic acid): placebos were the same color and shape as the active intervention drugs. Risk unclear | Placebos used. Not clear if e.g. study-code revealed treatment arm.  Low risk. | High risk: more than 20% loss to follow up | Women with severe anaemia (n=37) removed from study (“equally distributed among study groups”)  In main article, malaria outcome not reported. | No other obvious bias |

***Figure S1.2*: Quality assessment of observational studies (in alphabetical order)**

| **Author, year of publication and study design** | **Appropriate source population (selection bias)** | **Selection of participants (selection bias)** | **Appropriate tests (detection and information bias)** | **Incomplete exposure or outcome data (attrition bias)** | **Methods to control confounding (multivariate analysis)** | **Total sample size > 100** | **Malaria & iron primary (1) or secondary (2) analysis** | **Other potential bias detected** | **Notes** | **Assessment quality for review** |
| --- | --- | --- | --- | --- | --- | --- | --- | --- | --- | --- |
| Abrams 2005 [[7](#_ENREF_7)]  Case control | ⬣ | ⬣ | ⬣ | ⬣ | ▲ | ▲ | 2 | ⬣ |  | Good |
| Asaolu 2009 [[8](#_ENREF_8)]  Case-control | ⬣ | ▲ | ▲ | ▲ | ▲ | ⬣ | 1 | ⬣ |  | Low-to-Moderate |
| Ayoya 2006 [[9](#_ENREF_9)]  Cross-sectional | ⬣ | ⬣ | ⬣ | ▲ | ⬣ | ⬣ | 2 | ⬣ |  | Low-to-Moderate |
| Byles 1970 [[10](#_ENREF_10)] Prospective cohort | ⬣ | ⬣ | ⬣ | ▲ | ▲ | ⬣ | 1 | ▲ | Only malaria testing among participants with severe reactions | Low-to-Moderate |
| Danquah 2008 [[11](#_ENREF_11)]  Mockenhaupt 2000 [[12](#_ENREF_12)]  Cross-sectional | ⬣ | ▲ | ⬣ | ⬣ | ⬣ | ⬣ | 1 | ⬣ |  | Low-to-Moderate |
| Dreyfuss 2000 [[13](#_ENREF_13)]  Cross-sectional | ⬣ | ⬣ | ⬣ | ⬣ | ⬣ | ⬣ | 1 | ⬣ |  | Low-to-Moderate |
| Engmann 2008 [[14](#_ENREF_14)]  Cross-sectional | ⬣ | ⬣ | ⬣ | ⬣ | ⬣ | ⬣ | 1 | ▲ | Enrolment stratified by gestational age | Good |
| Eteng 2010 [[15](#_ENREF_15)]  Case-control | ⬣ | ▲ | ⬣ | ⬣ | ▲ | ▲ | 1 | ▲ | Sub group: 1^st^ trimester | Low-to-Moderate |
| Hinderaker 2002 [[16](#_ENREF_16)]  Case-Control | ⬣ | ⬣ | ⬣ | ⬣ | ▲ | ⬣ | 2 | ▲ | Case control for anaemia, not for exposure or outcome | Good |
| Huddle 1999 [[17](#_ENREF_17)]  Cross-sectional | ⬣ | ⬣ | ⬣ | ⬣ | ⬣ | ⬣ | 1 | ⬣ |  | Good |
| Kabyemela 2008 [[18](#_ENREF_18)]  Cross-sectional | ⬣ | ⬣ | ⬣ | 🞇 | ⬣ | ⬣ | 1 | ⬣ |  | Low-to-Moderate |
| Kapito-Tembo 2010 [[19](#_ENREF_19)] Cross-sectional | ⬣ | ⬣ | ⬣ | ⬣ | ⬣ | ⬣ | 1 | ⬣ | Sub group: HIV+ women | Good |
| Massawe 2002 [[20](#_ENREF_20)]  Cross-sectional | ⬣ | ⬣ | ⬣ | ⬣ | ▲ | ▲ | 2 | ⬣ | Sub group: Primigravidae < 21 | Good |
| Matteelli 1994 [[21](#_ENREF_21)]  Cross-sectional | ⬣ | ▲ | ⬣ | ▲ | ▲ | ⬣ | 2 | ⬣ |  | Low-to-Moderate |
| Mwapasa 2004 [[22](#_ENREF_22)]  Cross-sectional | ⬣ | ⬣ | ▲ | ⬣ | ⬣ | ⬣ | 2 | ⬣ |  | Low-to-Moderate |
| Nacher 2003 [[23](#_ENREF_23)]  Cohort | ⬣ | ⬣ | ⬣ | 🞇 | ⬣ | ⬣ | 1 | ⬣ |  | Low-to-Moderate |
| Ndyomugyenyi 2008 [[24](#_ENREF_24)] Cross-sectional | ⬣ | ⬣ | ⬣ | ⬣ | ⬣ | ⬣ | 1 | ▲ | Iron status by malaria not reported. Only p-value available | Good |
| Oppenheimer 1986 [[25](#_ENREF_25)]  Retrospective cohort | ⬣ | ▲ | 🞇 | ▲ | ▲ | ⬣ | 2 | ⬣ |  | Low-to-Moderate |
| Ouedraogo 2012 [[26](#_ENREF_26),[27](#_ENREF_27)]  Cross-sectional | ⬣ | ⬣ | ⬣ | ⬣ | ▲ | ⬣ | 2 | ▲ | Data part of IPTp trial  Subgroup: HIV(-) | Good |
| Reinhardt 1978 [[28](#_ENREF_28)]  Cross-sectional | ⬣ | ▲ | 🞇 | 🞇 | ▲ | ⬣ | 1 | ⬣ |  | Low-to-Moderate |
| Saad 2012 [[29](#_ENREF_29)]  Case-control | 🞇 | ▲ | ⬣ | ⬣ | ▲ | ▲ | 1 | ⬣ |  | Low-to-Moderate |
| Senga 2011 [[30](#_ENREF_30)]  Case control | ⬣ | ⬣ | ⬣ | ▲ | ⬣ | ⬣ | 1 | ⬣ |  | Low-to-Moderate |
| Senga 2012 [[31](#_ENREF_31)]  Cross-sectional | ⬣ | ⬣ | ⬣ | ⬣ | ▲ | ⬣ | 1 | ⬣ |  | Good |
| Shulman 1996 [[32](#_ENREF_32)]  Cross-sectional | ⬣ | ⬣ | ⬣ | ▲ | ▲ | ⬣ | 2 | ⬣ |  | Low-to-Moderate |
| Van Eijk 2007 [[33](#_ENREF_33)]  Cohort | ⬣ | ⬣ | ▲ | ⬣ | ⬣ | ⬣ | 2 | ⬣ |  | Low-to-Moderate |
| Van Santen 2011 [[34](#_ENREF_34)]  Cross-sectional | ⬣ | ⬣ | ⬣ | 🞇 | ▲ | ▲ | 1 | ⬣ | Sub group: Primigravidae | Low-to-Moderate |
| vanderJagt 2007 [[35](#_ENREF_35)]  Cross-sectional | ⬣ | ⬣ | ⬣ | ⬣ | ▲ | ⬣ | 2 | ⬣ |  | Good |

⬣: Adequate or appropriate. 🞇: Unclear from available information. ▲: Inappropriate or inadaequate.

***Table S1.2*: Observational studies quality assessment description**

| **Author/year, country of study** | **N** | **Study population and study design** | **Inclusion/exclusion criteria described and adequate for sample selection** | **Tests described and appropriate** | **Incomplete exposure/outcome data** | **Multivariate analysis for tests/markers** | **Other bias and notes** |
| --- | --- | --- | --- | --- | --- | --- | --- |
| Abrams 2005 [[7](#_ENREF_7)]  Malawi | 90 | Hospital based  Case control | Yes | Yes | Malaria and iron status for all participants | No | Possible (higher density parasitemia more likely to be enrolled as case than as control) |
| Asaolu 2009 [[8](#_ENREF_8)] Nigeria | 295 | ANC based  Case-control | No description | No description of malaria test | Only results for 70/195 malaria cases presented (36%). Only biomarker result for 160/295 (54%). No explanation given. | No |  |
| Ayoya 2006 [[9](#_ENREF_9)] Mali | 190 | ANC based  Cross-sectional | Yes | Yes | Only results for 131/190 (68.9%) | Multivariate analysis for low serum iron  (< 12 μmol/L) |  |
| Byles 1970 [[10](#_ENREF_10)]  Tanzania | 928 | Hospital based  Prospective cohort | Yes | Yes | Results for 917/928 (98.8%), and for all with generalized reaction, but no systematic malaria testing | No |  |
| Danquah 2008 [[11](#_ENREF_11)] Mockenhaupt 2000 [[12](#_ENREF_12)]  Ghana | 530 | ANC based  Cross-sectional | No.  Consecutive enrolment,  criteria not clear  Not clear if 1^st^ ANC visit | Yes | 527/530 iron status  530/530 malaria status | Yes |  |
| Dreyfuss 2000 [[13](#_ENREF_13)] Nepal | 336 | Clinic based  Cross-sectional | Yes | Yes | 336/336 iron status  288/336 malaria status (unreadable smears or started before malaria protocol implemented) | Yes | Potential participants recruited in the community had to visit the study clinic to be enrolled |
| Engmann 2008 [[14](#_ENREF_14)]  Ghana | 452 | ANC based  Cross-sectional  Stratified enrolment by gestational age | Yes | Yes | 428/452 included: “complete data” | Yes | Not enough information to stratify analysis by gestational age. |
| Eteng 2010 [[15](#_ENREF_15)]  Nigeria | 27 | ANC based  Case Control | No  According to discussion, only women in first trimester enrolled: not mentioned in enrolment criteria  Cases using iron or haematinics were excluded, but this did not seem to apply to control group | Yes | 27/27 malaria and iron biomarker data | No |  |
| Hinderaker 2002 [[16](#_ENREF_16)] | 312 | ANC based Case-control | Yes | Yes | 311/312 for iron status, 301/312 for malaria | No | Enrolment based on hemoglobin-strata. |
| Huddle 1999 [[17](#_ENREF_17)]  Malawi | 152 | ANC based  Cross-sectional | Yes | Yes | 142-150/152 for iron biomarker data | Yes | “Convenience sample” stated |
| Kabyemela 2008 [[18](#_ENREF_18)] Tanzania | 445 | Hospital based,  Cross-sectional | Yes  Consecutive enrolment and consent, further described in another article | Yes | Iron and malaria status for 445 women determined, but not clear what it could have been | Yes |  |
| Kapito-Tembo 2010 [[19](#_ENREF_19)]  Malawi | 455 | ANC based,  Cross-sectional | Yes | Iron use based on self-report | Iron status only for first 455/1142 enrolments  Malaria for 433/455  Iron supplementation for 1133/1142 | Yes |  |
| Massawe 2002 [[20](#_ENREF_20)] Tanzania | 76 | ANC based  Cross-sectional | Yes | Malaria testing not well described | 76/76 for both malaria and iron status | No |  |
| Matteelli 1994 [[21](#_ENREF_21)] Zanzibar | 440 | Hospital based,  Cross-sectional | No  Consecutive enrolment, uncomplicated delivery, not further described | Yes | 385/440 for malaria  267/440 for ferritin  394/440 for free serum iron | No |  |
| Mwapasa 2004 [[22](#_ENREF_22)] Malawi | 1662 | Hospital based,  Cross-sectional | Yes | Iron use based on self-report | Information available for all participants | No | Results by HIV-status, but no stratified enrolment |
| Nacher 2003 [[23](#_ENREF_23)]  Thailand | 2112 | ANC based  Cohort | Yes | Yes | No; no report on how much information was missing for malaria testing or iron supplementation information | Yes |  |
| Ndyomugyenyi 2008 [[24](#_ENREF_24)]  Uganda | 832 | ANC based  Cross-sectional | Yes | Yes | Ferritin for 817/834 and malaria for 802/834 | Yes | Only p-value multivariate analysis available |
| Oppenheimer 1986 [[25](#_ENREF_25)]  Papua New Guinea | 544 | Hospital  Retrospective cohort | No description of inclusion/exclusion criteria or enrolment procedures | No | Malaria and total dose iron results for 364/544 (66.9%), no explanation | No | Malaria causes anemia, therefore, women receiving TDI (Hb<8 g/dl) were more likely to be infected with malaria |
| Ouedraogo 2012 [[26](#_ENREF_26),[27](#_ENREF_27)] | 1005  868 | ANC based  Cohort | Yes | Yes | No | No | Part of a trial into IPTp for MiP |
| Reinhardt 1978 [[28](#_ENREF_28)] Cote d’Ivoire | 198 | Hospital  Cross-sectional | No description of inclusion/exclusion criteria or enrolment procedures | No description of tests in this manuscript | Not clear how many enrolled and for how many results were available | No | For laboratory tests referred to other manuscript |
| Saad 2012 [[29](#_ENREF_29)]  Sudan | 96 | Hospital  Case-control | No good description on how controls were identified and enrolled | Yes | 96/96 for malaria and ferritin | No | Matched for age, parity, gestational age and weight |
| Senga 2011 [[30](#_ENREF_30)]  Malawi | 222 | Hospital Based  Case-control | Yes | Yes | 160/222 for iron status (72.1%)  222/222 for malaria | Yes |  |
| Senga 2012 [[31](#_ENREF_31)]  Malawi | 4103  1327 | ANC  Cohort | Yes | Yes | Iron status for 3970/4103 (pregnancy) and 1327/1327 (delivery)  Malaria status for all | Yes |  |
| Shulman 1996 [[32](#_ENREF_32)] Kenya | 275 | ANC  Cross-sectional | Yes | Yes | 217/275 serum ferritin (78.9%)  275/275 malaria status | No |  |
| Van Eijk 2007 [[33](#_ENREF_33)] Kenya | 3108 | ANC  Cohort | Yes | No verification of intake haematinics | Malaria status for all | Yes | Results by HIV-status, but no stratified enrolment |
| Van Santen 2011 [[34](#_ENREF_34)] Gabon | 69 | Hospital  Cross-sectional | Yes | Yes | Not clear (unknown how many women with potential information) | No |  |
| vanderJagt 2007 [[35](#_ENREF_35)] Nigeria | 146 | ANC  Cross-sectional | Yes | Yes | Malaria and iron status for all participants available | No |  |

***Supporting information section 2*: Supplementary graphs on malaria and iron supplementation in pregnancy**

***Figure S2.1*: Sub-group analysis malaria among iron-supplemented and non-supplemented pregnant women: HIV-status**

**
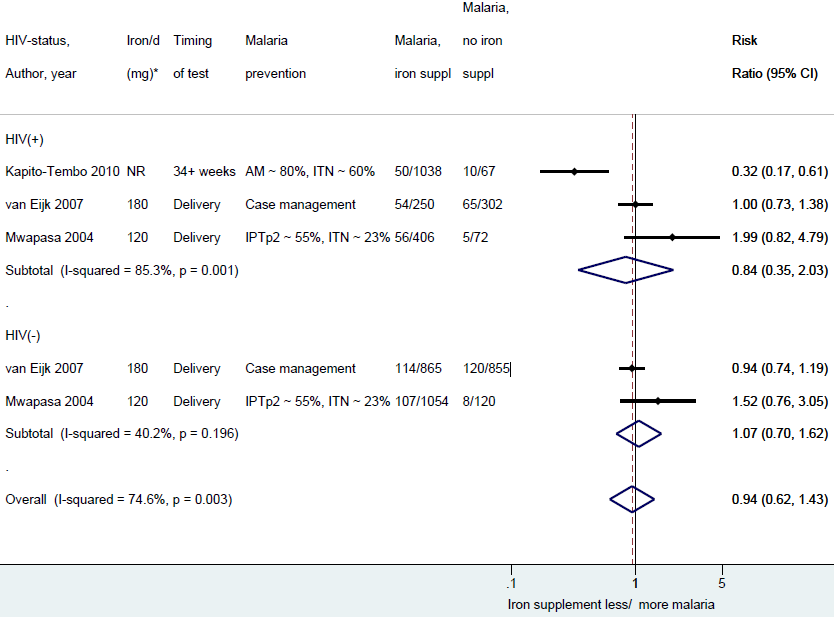
**

BS: blood smear. CI: confidence interval. HIV: human immunodeficiency virus.

Sub-group analysis comparing HIV(+) vs. HIV(-): p=0.6

***Figure S2.2*: Sub-group analysis malaria among iron-supplemented and non-supplemented pregnant women: Sickle cell trait**

**
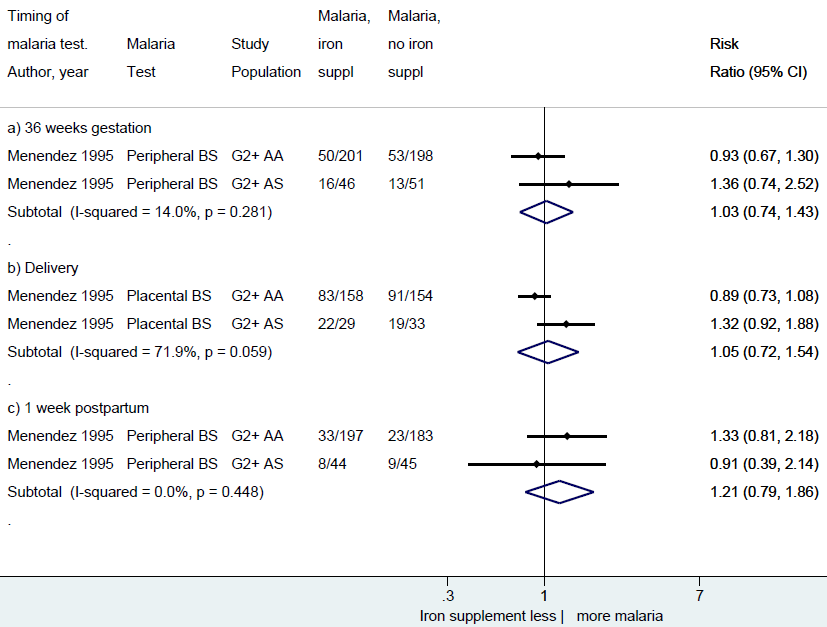
**

AA: haemoglobin genotype AA, no sickle cell gene. AS: haemoglobin genotype AS, 1 sickle cell gene. BS: bloodsmear. CI: confidence interval. G2+: multigravidae.

Subgroup analysis: comparing AA and AS at 36 weeks gestation: p=0.3; comparing AA and AS at delivery: p=0.06, comparing AA and AS postpartum: p=0.4

***Figure S2.3*: Sub-group analysis malaria among iron-supplemented and non-supplemented pregnant women: duration of iron supplementation**

**
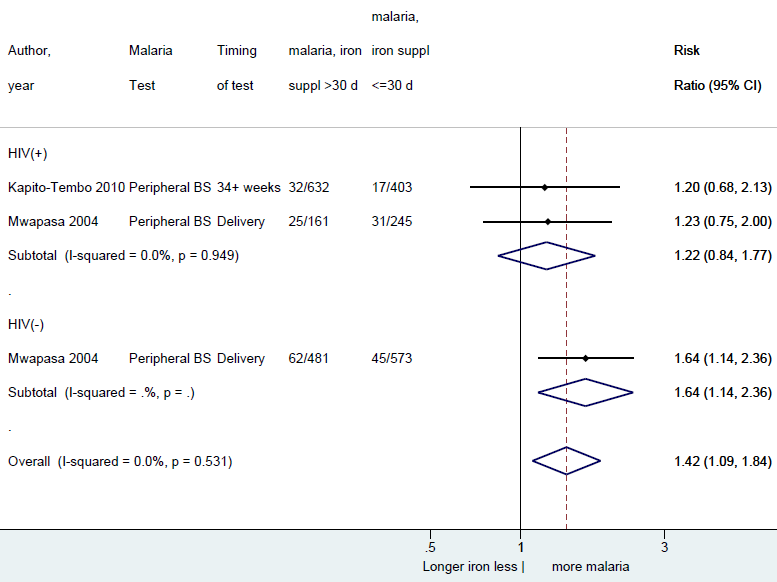
**

BS: blood smear. CI: confidence interval.

Subgroup analysis: comparing HIV(+) vs. HIV(-): p=0.3

Note that both these studies are surveys with retrospective history on iron use. In both studies, iron supplementation was associated with the intake of sulfadoxine-pyrimethamine (SP) [[22](#_ENREF_22)], or SP and cotrimoxazole, or antiretroviral treatment [[19](#_ENREF_19)].

***Supporting information section 3*: Supplementary graphs on malaria and iron deficiency in pregnancy**

***Figure S3.1*: Listing of additional definitions used for iron deficiency, and malaria risk among iron deficient and not-iron deficient women, sub-Saharan Africa and Asia**

**
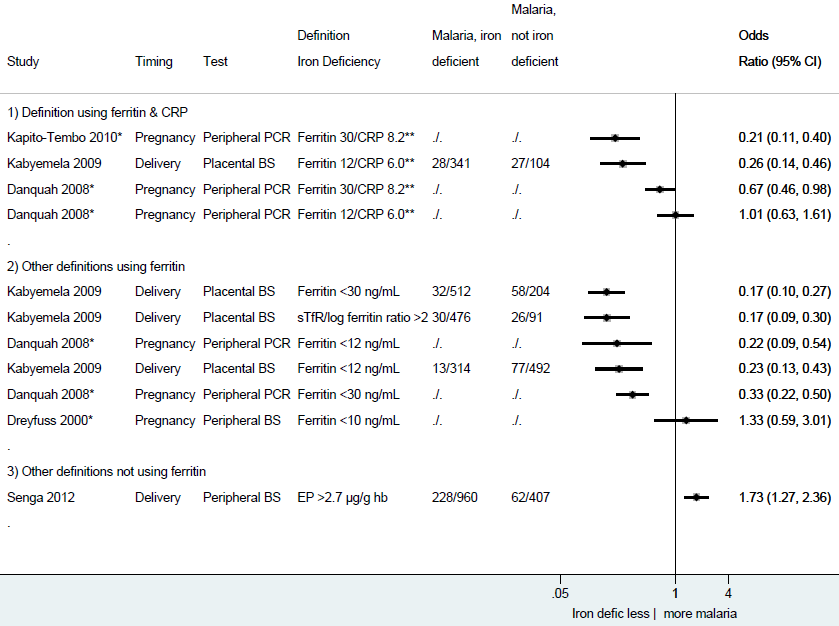
**

BS: blood smear. CI: confidence interval. CRP: C-reactive protein. EP: Erythrocyte protoporphyrin, PCR: polymerase chain reaction. sTfR: Soluble transferrin receptor.

*Adjusted odds ratios used: Kapito-Tembo 2010: odds ratio adjusted for CD4 count, gravidity, intestinal infections and PCR, only HIV(+) women included.

Danquah 2008: odds ratio adjusted for gravidity and chloroquine or pyrimethamine detectable in urine

Dreyfuss 2000: odds ratio adjusted for hookworm infection, serum retinol and trimester of pregnancy

** Ferritin 30/CRP 8.2: Ferritin <30 ng/mL & CRP <=8.2 ng/mL or ferritin <70 ng/mL & CRP >8.2 ng/mL

Ferritin 12/CRP 6.0: Ferritin <12 ng/ml & CRP <6 ng/ml or ferritin <=50 ng/ml & CRP >= 6 ng/ml

***Figure S3.2*: Subgroup analysis by gravidity for malaria risk among iron deficient and not-iron deficient women, sub-Saharan**

**Africa**


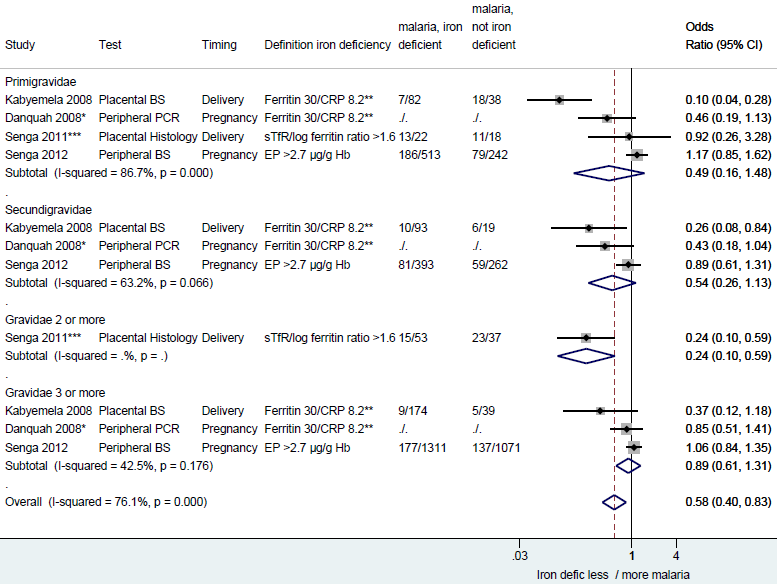


BS: blood smear. CI: confidence interval. CRP: C-reactive protein. EP: Erythrocyte protoporphyrin. PCR: polymerase chain reaction. sTfR: Soluble transferrin receptor.

*For Danquah 2008 no raw numbers available

** Ferritin 30/CRP 8.3: Ferritin <30 ng/mL & CRP <=8.2 ng/mL or ferritin <70 ng/mL & CRP >8.2 ng/mL

***Senga 2011 Placental histology: Active infection defined by acute or chronic infection (parasites alone, or parasites in the presence of hemozoin). No placental infection defined by the absence of parasites and hemozoin

Subgroup analysis comparing gravidity groups: p=0.6. The grey block around each estimate represents the weight of the study.

***Supporting information section 4*: Supplementary data on iron supplementation, iron status and malaria risk in pregnancy**

***Table S4.1*: Iron Supplementation vs. Malaria Risk: additional outcomes not presented in forest plots**

| **Author, Year** | **Study Design** | **Study Population** | **Type & Timing of malaria test (or supplementation)** | **Exposed or Cases**  **% Outcome (n)** | **Comparison**  **% Outcome (n)** | **Risk estimate**  **(95% CI)** |
| --- | --- | --- | --- | --- | --- | --- |
| **Outcome: Peripheral parasitemia** | | | | | | |
| Kapito-Tembo^1^,  2010 [[19](#_ENREF_19)] | Cross-sectional | HIV (+) | Peripheral PCR in  3^rd^ trimester | Any iron use  9·3% (98/1051) | No iron use  19·1% (13/68) | OR 0·43 (0·23-0·83)  aOR^1^ 0·70 (0·30-1·64)  RR 0·49 (0·29, 0·82) |
| Menendez, 1994 [[4](#_ENREF_4)] | RCT | All | Peripheral BS  in postpartum | Iron  17·3% (43/249) | Placebo  13·2% (32/242) | RR 1·31 (0·86, 1·99) |
| Mwapasa,  2004 [[22](#_ENREF_22)] | Cross-sectional | All | Peripheral BS  at delivery | Iron use >30 days  13·6% (87/642) | Iron use ≤30 days  9·2% (76/823) | OR 1·54 (1·11-2·14)  RR 1·47 (1·10-1·96) |
| Ndyomugyenyi,  2000 [[6](#_ENREF_6)] | RCT | All | Peripheral BS  at delivery | CM + Iron/FA  40·0% (62/155) | CM + Placebo  49·0% (76/155) | RR 0·82 (0·63, 1·05) |
| van Eijk,  2007 [[33](#_ENREF_33)] | Before-After study | All | Peripheral BS  at delivery | Period 2:  Hematinic supplementation  Sept 97 - March 99  15·1% (168/1115) | Period 1:  No intervention  Before Sept 1997  16·0% (185/1157) | RR 0·94 (0·78, 1·14) |
| **Outcome: Placental malaria** | | | | | | |
| Menendez, 1994 [[4](#_ENREF_4)] | RCT | All | Placental histology – Active or Chronic | Iron  21% (40/195) | Placebo  19% (37/198) | RR 1·06 (0·83, 1·35) |
| Menendez, 1994 [[4](#_ENREF_4)] | RCT | All | Placental BS  at delivery | Iron  8·9% (17/191) | Placebo  8·4% (15/179) | RR 1·06 (0·55, 2·06) |
| van Eijk, 2007 [[33](#_ENREF_33)] | Before-After study | HIV (-) | Placental BS  at delivery | Period 2:  Hematinic supplementation  Sept 97 - March 99  16% (142/879) | Period 1:  No intervention  before Sept 1997  16% (136/852) | RR 1·01 (0·82, 1·26)  OR 1·01 (0·78-1·31)  aOR^2^ 1·01 (0·78-1·31) |
| van Eijk, 2007 [[33](#_ENREF_33)] | Before-After study | HIV (+) | Placental BS  at delivery | Period 2:  Hematinic supplementation  Sept 97 - March 99  29% (75/256) | Period 1:  No intervention  before Sept 1997  25% (75/303) | RR 1·18 (0·9, 1·56)  OR 1·26 (0·87-1·83)  aOR^2^ 1·21 (0·67-2·19) |

BS: Blood slide RR: Risk Ratio. OR: Odds ratios. aOR: adjusted Odds ratios.

Kapito-Tembo 2010 & Mwapasa 2004 – results originally presented as Odds Ratios

^1^ Adjusted for age, gravidity, bed net use, socio-economic status, antenatal visits, use of intermittent preventive treatment with sulfadoxine-pyrimethamine and cotrimoxazole prophylaxis

^2^ Adjusted for gravidity, HIV, interaction term HIV and gravidity, age, place of residence, ethnicity and socio-economic status

***Table S4.2*: Iron Deficiency vs. Malaria Risk: additional outcomes not presented in forest plots**

| **Author, Year** | **Study Design** | **Outcome**  **(Type & Timing of malaria test or iron deficiency)** | **Exposed or Cases**  **% Outcome (n)** | **Comparison**  **% Outcome (n)** | **Odds Ratio (95% CI)**  **or p-value** |
| --- | --- | --- | --- | --- | --- |
| Senga, 2012 [[31](#_ENREF_31)] | Cross-sectional | Peripheral BS 1^st^ ANC visit | Iron deficiency^1^  20.0 (444/2218) | No iron deficiency  17·5 (276/1575) | RR 1·14 (1·00-1·31)  OR 1·18 (1·00-1·39) |
| Senga, 2012 [[31](#_ENREF_31)] | Cross-sectional | Peripheral BS delivery | Iron deficiency^1^  23·8 (228/960) | No iron deficiency  15·2 (62/407) | RR 1·56 (1·21-2·02)  OR 1·73 (1·27-2·36) |
| Senga, 2012 [[31](#_ENREF_31)] | Cross-sectional | Placental BS delivery | Iron deficiency^1^  19·0 (178/935) | No iron deficiency  15·6 (61/390) | RR 1·22 (0·93-1·59)  OR 1·27 (0·92-1·74) |
| **Iron Deficiency stratified by gravidity** | | | | | |
| Senga, 2012 [[31](#_ENREF_31)] | Cross-sectional | Peripheral BS delivery-PG | Iron deficiency^1^  38·7% (84/217) | No iron deficiency  26·8% (22/82) | RR 1·44 (0·97-2·14)  OR 1·72 (0·98-3·01) |
| Senga, 2012 [[31](#_ENREF_31)] | Cross-sectional | Peripheral BS delivery-SG | Iron deficiency^1^  26·4% (43/163) | No iron deficiency  20·6% (13/63) | RR 1·28 (0·74-2·21)  OR 1·38 (0·68-2·78) |
| Senga, 2012 [[31](#_ENREF_31)] | Cross-sectional | Peripheral BS delivery-MG | Iron deficiency^1^  17·4% (101/580) | No iron deficiency  10·3% (27/262) | RR 1·69 (1·13-2·52)  OR 1·84 (1·17-2·89) |
| Senga, 2012 [[31](#_ENREF_31)] | Cross-sectional | Placental BS delivery-PG | Iron deficiency^1^  34·6% (73/211) | No iron deficiency  31·3% (25/80) | RR: 1·11 (0·76-1·61)  OR: 1·16 (0·67-2·02) |
| Senga, 2012 [[31](#_ENREF_31)] | Cross-sectional | Placental BS delivery-SG | Iron deficiency^1^  21·8% (34/156) | No iron deficiency  18·2% (10/55) | RR: 1·20 (0·64-2·26)  OR: 1·25 (0·57-2·75) |
| Senga, 2012 [[31](#_ENREF_31)] | Cross-sectional | Placental BS delivery-MG | Iron deficiency^1^  12·5% (71/568) | No iron deficiency  10·2% (26/255) | RR: 1·23 (0·80-1·87)  OR: 1·26 (0·78-2·02) |

aOR: adjusted Odds Ratio. BS: Blood slide. PCR: Polymerase Chain reaction. PF: *Plasmodium falciparum*. PG: primigravidae. PV: *Plasmodium vivax.* MG: multigravidae. OR: Odds ratio. RR: Risk ratio. SG: secundigravidae.

**Definitions of iron deficiency or placental infection:**

^1^Red cell zinc protoporphyrin/haem > 2.7 μg/g hemoglobin

***Supporting information section 5*: Effect of iron supplementation on hemoglobin or anemia in pregnancy in iron supplementation studies included in this review**

***Figure S5.1*: The effect of iron supplementation on anemia in pregnancy in studies involved in this review (random effects analysis)**


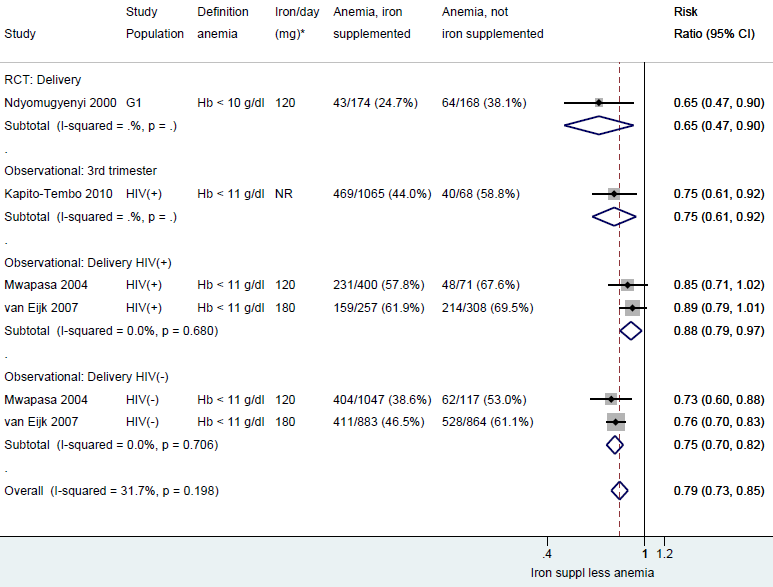


CI: confidence interval. Hb: hemoglobin. G1: primigravidae. RCT: randomized controlled trial. Suppl: supplementation.

The grey areas around the risk ratios indicate the study weight.

***Figure S5.2*: The effect of iron supplementation on hemoglobin in pregnancy in studies involved in this review (random effects analysis)**


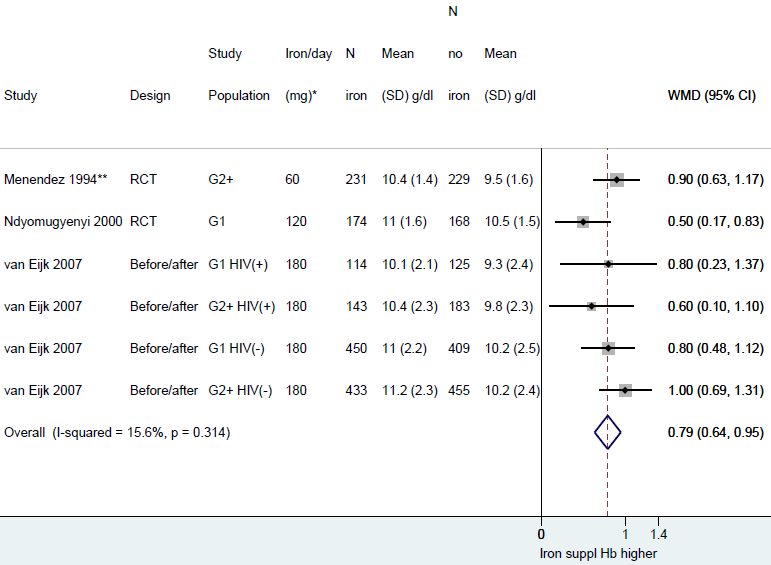


CI: confidence interval. G1: primigravidae. G2+: multigravidae. N: sample size. RCT: randomized controlled trial. SD: standard deviation. WMD: weighted mean difference.

*Daily elementary iron dose

** Mean hemoglobin in third trimester in Menendez 1994. All other studies use mean hemoglobin at the time of delivery.

The grey areas around the mean difference indicate the study weight.

**Supporting information references**

1. Higgins JP, Altman DG, Gotzsche PC, Juni P, Moher D, et al. (2011) The Cochrane Collaboration's tool for assessing risk of bias in randomised trials. BMJ 343: d5928.

2. Sanderson S, Tatt ID, Higgins JP (2007) Tools for assessing quality and susceptibility to bias in observational studies in epidemiology: a systematic review and annotated bibliography. Int J Epidemiol 36: 666-676.

3. von Elm E, Altman DG, Egger M, Pocock SJ, Gotzsche PC, et al. (2007) The Strengthening the Reporting of Observational Studies in Epidemiology (STROBE) statement: guidelines for reporting observational studies. Bull World Health Organ 85: 867-872.

4. Menendez C, Todd J, Alonso PL, Francis N, Lulat S, et al. (1994) The effects of iron supplementation during pregnancy, given by traditional birth attendants, on the prevalence of anaemia and malaria. Trans R Soc Trop Med Hyg 88: 590-593.

5. Menendez C, Todd J, Alonso PL, Francis N, Lulat S, et al. (1995) The response to iron supplementation of pregnant women with the haemoglobin genotype AA or AS. Trans R Soc Trop Med Hyg 89: 289-292.

6. Ndyomugyenyi R, Magnussen P (2000) Chloroquine prophylaxis, iron/folic-acid supplementation or case management of malaria attacks in primigravidae in western Uganda: effects on congenital malaria and infant haemoglobin concentrations. Ann Trop Med Parasitol 94: 759-768; discussion 769-770.

7. Abrams ET, Kwiek JJ, Mwapasa V, Kamwendo DD, Tadesse E, et al. (2005) Malaria during pregnancy and foetal haematological status in Blantyre, Malawi. Malar J 4.

8. Asalou MF, Igbaakin PA (2009) Serum Levels of micronutrients and antioxidants during malaria in pregnant women In Ado-Ekiti, Ekiti State, Nigeria. International Journal of Medicine and Medical Sciences 1: 523-526.

9. Ayoya MA, Spiekermann-Brouwer GM, Traore AK, Stoltzfus RJ, Garza C (2006) Determinants of anemia among pregnant women in Mali. Food Nutr Bull 27: 3-11.

10. Byles AB, D'sa A (1970) Reduction of reaction due to iron dextran infusion using chloroquine. Br Med J 3: 625-627.

11. Danquah I, Bedu-Addo G, Mockenhaupt FP (2008) Iron deficiency and Plasmodium falciparum infection during pregnancy. J Infect Dis 198: 1573-1574.

12. Mockenhaupt FP, Rong B, Gunther M, Beck S, Till H, et al. (2000) Anaemia in pregnant Ghanaian women: importance of malaria, iron deficiency, and haemoglobinopathies. Trans R Soc Trop Med Hyg 94: 477-483.

13. Dreyfuss ML, Stoltzfus RJ, Shrestha JB, Pradhan EK, LeClerq SC, et al. (2000) Hookworms, malaria and vitamin A deficiency contribute to anemia and iron deficiency among pregnant women in the plains of Nepal. J Nutr 130: 2527-2536.

14. Engmann C, Adanu R, Lu TS, Bose C, Lozoff B (2008) Anemia and iron deficiency in pregnant Ghanaian women from urban areas. Int J Gynaecol Obstet 101: 62-66.

15. Eteng MU, Ekwe AO, Eyong EU, Ibekwe HA, Abolaji AO, et al. (2010) Biochemical and haematological changes in pregnant malaria patients and pregnant non-malaria women. Scientific Research and Essays 59: 1009-1013.

16. Hinderaker SG, Olsen BE, Lie RT, Bergsjo PB, Gasheka P, et al. (2002) Anemia in pregnancy in rural Tanzania: associations with micronutrients status and infections. Eur J Clin Nutr 56: 192-199.

17. Huddle JM, Gibson RS, Cullinan TR (1999) The impact of malarial infection and diet on the anaemia status of rural pregnant Malawian women. Eur J Clin Nutr 53: 792-801.

18. Kabyemela ER, Fried M, Kurtis JD, Mutabingwa TK, Duffy PE (2008) Decreased susceptibility to Plasmodium falciparum infection in pregnant women with iron deficiency. J Infect Dis 198: 163-166.

19. Kapito-Tembo A (2010) Malaria and anemia in HIV-infected pregnant women in Malawi: Associations with cotrimoxazole prophylaxis, submicroscopic malaria, iron supplementation and iron deficiency: Thesis. University of North Carolina at Chapel Hill. 135 p.

20. Massawe SN, Ronquist G, Nystrom L, Lindmark G (2002) Iron status and iron deficiency anaemia in adolescents in a Tanzanian suburban area. Gynecol Obstet Invest 54: 137-144.

21. Matteelli A, Donato F, Shein A, Muchi JA, Leopardi O, et al. (1994) Malaria and anaemia in pregnant women in urban Zanzibar, Tanzania. Ann Trop Med Parasitol 88: 475-483.

22. Mwapasa V (2004) The interactions between Plasmodium Falciparum malaria and HIV-1 in pregnant Malawian women: Thesis. The University of Michigan. 155 p.

23. Nacher M, McGready R, Stepniewska K, Cho T, Looareesuwan S, et al. (2003) Haematinic treatment of anaemia increases the risk of Plasmodium vivax malaria in pregnancy. Trans R Soc Trop Med Hyg 97: 273-276.

24. Ndyomugyenyi R, Kabatereine N, Olsen A, Magnussen P (2008) Malaria and hookworm infections in relation to haemoglobin and serum ferritin levels in pregnancy in Masindi district, western Uganda. Trans R Soc Trop Med Hyg 102: 130-136.

25. Oppenheimer SJ, Macfarlane SB, Moody JB, Harrison C (1986) Total dose iron infusion, malaria and pregnancy in Papua New Guinea. Trans R Soc Trop Med Hyg 80: 818-822.

26. Ouedraogo S, Koura GK, Accrombessi MM, Bodeau-Livinec F, Massougbodji A, et al. (2012) Maternal anemia at first antenatal visit: prevalence and risk factors in a malaria-endemic area in Benin. Am J Trop Med Hyg 87: 418-424.

27. Ouedraogo S, Bodeau-Livinec F, Briand V, Huynh BT, Koura GK, et al. (2012) Malaria and gravidity interact to modify maternal haemoglobin concentrations during pregnancy. Malar J 11: 348.

28. Reinhardt MC (1978) Maternal anaemia in Abidjan--Its influence on placenta and newborns. Helv Paediatr Acta Suppl: 43-63.

29. Saad AA, Mohamed OE, Ali AA, Bashir AM, Ali NI, et al. (2012) Acute-phase proteins in pregnant Sudanese women with severe Plasmodium falciparum malaria. Trans R Soc Trop Med Hyg 106: 570-572.

30. Senga EL, Harper G, Koshy G, Kazembe PN, Brabin BJ (2011) Reduced risk for placental malaria in iron deficient women. Malar J 10: 47.

31. Senga EL, Koshy G, Brabin BJ (2012) Zinc erythrocyte protoporphyrin as marker of malaria risk in pregnancy - a retrospective cross-sectional and longitudinal study. Malar J 11: 249.

32. Shulman CE, Graham WJ, Jilo H, Lowe BS, New L, et al. (1996) Malaria is an important cause of anaemia in primigravidae: evidence from a district hospital in coastal Kenya. Trans R Soc Trop Med Hyg 90: 535-539.

33. van Eijk AM, Ayisi JG, Slutsker L, Ter Kuile FO, Rosen DH, et al. (2007) Effect of haematinic supplementation and malaria prevention on maternal anaemia and malaria in western Kenya. Trop Med Int Health 12: 342-352.

34. Van Santen S, de Mast Q, Luty AJ, Wiegerinck ET, Van der Ven AJ, et al. (2011) Iron homeostasis in mother and child during placental malaria infection. Am J Trop Med Hyg 84: 148-151.

35. VanderJagt DJ, Brock HS, Melah GS, El-Nafaty AU, Crossey MJ, et al. (2007) Nutritional factors associated with anaemia in pregnant women in northern Nigeria. J Health Popul Nutr 25: 75-81.
